# The economic impact of deaths averted in PEPFAR countries

**DOI:** 10.1136/bmjgh-2025-022885

**Published:** 2026-11-11

**Authors:** William Crown, Deborah V Stenoien, Michael J A Reid, V S Senthil Kumar, Jennifer Kates, Monica Jordan, Allyala Nandakumar

**Affiliations:** 1 Center for Innovation in Social Work and Health, School of Social Work, Boston University, Boston, Massachusetts, USA; 2 Institute for Global Health Sciences, University of California San Francisco, San Francisco, California, USA; 3 Institute for Global Health and Development, Brandeis University Heller School for Social Policy and Management, Waltham, Massachusetts, USA; 4 Global Health and HIV Policy, The Kaiser Family Foundation, Washington, District of Columbia, USA

**Keywords:** HIV, Public Health, Health economics, Global Health

## Abstract

**Introduction:**

Previous research has demonstrated the lifesaving impact of the US President’s Emergency Plan for AIDS Relief (PEPFAR) programme, as well as the broader economic returns of global health investments more generally. As policymakers consider PEPFAR’s future, it is important to understand the programme’s wider benefits. This study builds on prior estimates of PEPFAR’s effect on reducing all-cause mortality (ACM), translates these reductions in premature deaths to estimate the total number of deaths averted, then analyses the associated impact on gross domestic product (GDP) in PEPFAR countries.

**Methods:**

This study uses a staggered difference-in-difference model with heterogeneous treatment effects to estimate the relationship between PEPFAR spending and ACM over time. A generalised method of moments system estimation model is then used to estimate the impact of ACM reductions on GDP in 30 PEPFAR countries.

**Results:**

There is a significant positive relationship between PEPFAR and reductions in ACM, with an average ACM reduction of 3.50 per 1000 people in PEPFAR countries from 2004 to 2022. These reductions translate to roughly 84.58 million premature deaths averted. The generalised method of moments system estimation model estimates that each one-point decrease in ACM is associated with a 4.9% increase in average GDP. Together, these estimates indicate that PEPFAR is associated with an additional 1.4% annual GDP growth in PEPFAR countries—for a total economic benefit of US$836 billion across countries from 2004 to 2022.

**Conclusion:**

This analysis finds that PEPFAR is associated with significant reductions in ACM that produce substantial economic gains. Disinvestment in the programme could result in forgoing future health and economic benefits.

**WHAT IS ALREADY KNOWN ON THIS TOPIC**

- Previous research has shown significant positive spillover effects from the US President’s Emergency Plan for AIDS Relief (PEPFAR), the US global HIV programme.
- While the programme aims to provide prevention, testing and treatment for HIV services, previous research has shown that countries that receive PEPFAR support are associated with improved maternal and child health outcomes, higher immunisation rates, increased school attendance and reductions in all-cause mortality (ACM).

**WHAT THIS STUDY ADDS**

- The present analysis expands upon previous studies by showing the time-varying impact of PEPFAR’s ACM reductions, translates these findings to estimate the total number of premature deaths averted and explores the impact of these averted deaths on gross domestic product (GDP) growth.
- The analysis shows that the PEPFAR programme is associated with an average ACM reduction of 3.50 per 1000 people, which translates to roughly 84.58 million deaths averted between 2004 and 2022.
- This estimate is significantly higher than the estimated 26 million lives saved, typically attributed to PEPFAR, when looking at reductions in HIV-related deaths alone.
- The present analysis estimates each one-point decrease in ACM to be associated with an annual 1.4% GDP increase in PEPFAR countries—for a total economic benefit of US$836 billion across countries from 2004 to 2022.

**HOW THIS STUDY MIGHT AFFECT RESEARCH, PRACTICE OR POLICY**

- The present analysis supports previous research and adds to the body of evidence on the significant positive spillover effects of PEPFAR on health and economic outcomes.
- These studies reaffirm the role of the PEPFAR programme as a strategic investment that has significant measurable impacts beyond health alone.
- Amidst current uncertainties over the programme’s future, these findings illuminate the scale of the programme’s health and economic benefits and reaffirm the importance of continued investment into the programme.

## Background

 US President’s Emergency Plan for AIDS Relief (PEPFAR), the US global HIV programme launched in 2003, remains the largest public health initiative ever dedicated to combating a single disease. Credited with saving more than 26 million lives, it has provided HIV prevention, testing and treatment services to millions of people, reduced new HIV infections and helped to build and strengthen local health systems.^1^

A foreign aid review that is underway by the Trump administration has resulted in pausing and discontinuing many PEPFAR services, and has led to significant service disruptions and uncertainties regarding PEPFAR’s future.^2^ A recent modelling analysis for South Africa, for example, estimated that reducing PEPFAR spending by 50% or 100% would result in an additional 286 000 and 565 000 HIV infections, respectively, over the next 10 years. People living with HIV would lose 2.02 and 3.71 life years under the two scenarios.^3^ In the face of these uncertainties, understanding PEPFAR’s broader health and economic impact would assist in assessing what gains may be at risk.

The positive economic spillovers from large scale health investments have been well documented.^4–9^ For example, based on economic theory reductions in mortality and morbidity in a population are expected to have positive effects on labour supply, directly impacting the productive capacity of an economy. Improved population health would be expected to result in greater labour productivity and improvements in school attendance from less absenteeism. Rising average life expectancy is expected to create incentives for investment in human capital and savings.^10^ Over the longer term, improvements in school attendance and investments in human capital are expected to contribute to additional gains in labour force productivity.^11^ Salaries paid to health professionals, construction workers, clinic and other healthcare staff circulate within the economy, creating a Keynesian multiplier effect that boosts spending, job creation and overall economic growth.^12^ With an investment of over US$120 billion in the programme, it is expected that the economic impacts of PEPFAR could be substantial.^13^

To date, a small but growing number of studies have examined the ‘spillover’ effects of PEPFAR—that is, the positive impacts beyond HIV. Gaumer *et al* found that PEPFAR has contributed to significant reductions in all-cause mortality (ACM), maternal and child mortality, and increases in childhood immunisation rates.^6 7^ Previous research has found that PEPFAR investments contributed positively to gross domestic product (GDP) growth rates,^14^ and that GDP per capita and productivity per worker were positively correlated with the level of PEPFAR investments.^15^ More recently, Crown *et al*^5^ found positive PEPFAR spillover effects on boys’ and girls’ school retainment rates and GDP per capita growth rates.

The present study seeks to expand on previous work in several ways. First, Gaumer *et al* estimate PEPFAR’s impacts on ACM over three different time periods—2004–2008, 2004–2013 and 2004–2018 and the results suggest that PEPFAR’s overall impact on ACM is increasing over time, although at a diminishing rate. First, we expand the time with four additional years of data (2019–2022) and use statistical methods that enable us to estimate heterogeneous treatment effects that provide direct estimates of how PEPFAR’s impacts on ACM have changed over time. Second, we apply the ACM reductions to total populations to estimate the number of premature deaths averted over this period. Third, we estimate the effects of ACM on GDP, then combine these estimates with those of PEPFAR’s incremental impact on ACM to arrive at an estimate of PEPFAR’s impact on GDP growth rate.

## Methods

We analyse panel data covering 157 low- and middle-income countries from 1990 to 2022 to assess the relationship between PEPFAR’s spending on ACM over time, as well as the related impacts of these ACM changes on the GDP of PEPFAR countries. Building on prior research by Gaumer *et al*^6 7^ and Crown *et al*^5^ this analysis expands the dataset, refines methodological approaches and applies additional robustness checks to deepen our understanding of PEPFAR’s effects. While the full data set includes 90 PEPFAR-supported countries, we focused on 30 present or former ‘COP’ countries, those that engaged in more intensive planning and programming through the preparation of ‘Country Operational Plans’ that document planned investments linked to results between 2004–2022. The comparison group includes 67 countries that received either no or minimal PEPFAR support (<US$1M or <US$0.05 per capita) during this same period (see online supplemental file S8). Our results exclude India from the PEPFAR cohort. Excluding India had virtually no effect on the model estimation results because of the relatively small number of observations it contributed. However, its large population size would have skewed the estimated number of deaths averted substantially and distorted the estimates for COP countries as a group. Results including India were conducted as a sensitivity analysis and are reported in the online supplemental file S5. The dataset includes information on ACM, per-capita GDP and country characteristics such as urban population, education, per-capita spending on health by PEPFAR, other donor programmes and domestic spending. A complete list of variables and their sources is provided in table 1. This is an updated version of the dataset and a similar set of covariates used in previous studies by Gaumer *et al* and Crown.^5–7^ All analyses were performed in accordance with Strengthening the Reporting of Observational Studies in Epidemiology (STROBE) guidelines for observational studies.^16^

**Table 1:** Baseline variables

Variable	Data source
GDP per capita (current USD)	World Bank Development Indicators ^33^
Recipient of US HIV funding prior to 2004 (dummy variable)	USAID ^34^
Total population	United Nations, Department of Economic and Social Affairs, Population Division ^35^
Life expectancy at birth (years)	World Bank Development Indicators ^33^
Total fertility rate (births per woman)	World Bank Development Indicators ^33^
Per cent urban population (of total population)	World Bank Development Indicators ^33^
School enrolment, secondary (% gross)	World Bank Development Indicators ^33^
World Bank country income classification	World Bank ^33^
HIV prevalence (% of population ages 15–49)	World Bank Development Indicators^33^ To address missing values in some cases, additional data were obtained from the Global Burden of Disease Collaborative Network^36^
Per capita donor spending on health (non-PEPFAR) (constant USD)	OECD Creditor Reporting System database ^37^
Per capita domestic health spending, government and private, PPP (current USD)	World Bank Development Indicators ^33^

Source. –

GDP, gross domestic product; OCED, Organisation for Economic Cooperation and Development; PEPFAR, US President’s Emergency Plan for AIDS Relief; PPP, purchasing power parity; USAID, US Agency for International Development; USD, US dollars.

Difference-in-difference (DID) methods have been an active area of research in econometrics literature.^17–19^ The common DID model estimates three values of interest: (1) a time dummy variable to demonstrate overall differences in mean values of the ACM rate between the baseline period and the follow-up period for the comparison group, (2) a dummy variable to represent differences in the intervention and comparison groups prior to programme implementation and (3) the coefficient on the variables representing the interaction between PEPFAR and the time dummy variable to measure the impact of the PEPFAR programme.^20^

We use panel data with DID methods to compare ACM in PEPFAR COP countries to the comparison group. We then measured ACM in the follow-up period in both groups, controlling for unobserved characteristics within countries that may impact outcomes but remain fixed over time.

We use an extension of the common DID model using panel data methods that address the potential heterogeneity of PEPFAR impacts over time (Stata xthdidreg). We then apply the average annual ACM reduction to the population by year to estimate the number of deaths averted. See online supplemental files S1–S3 for additional detail.

We also use panel data methods to model the related impacts of ACM on GDP, controlling for other variables available in the data. Estimating the relationship between ACM and GDP is complex as each may influence the other over time. We use generalised method of moments (GMM) system estimation (Stata xtabond2) to adjust for the potential endogeneity of ACM and GDP over time. The methods are described in more detail below.

We used the panel data statistical methods described above to estimate the impacts of PEPFAR on ACM and then separately estimate the relationship between ACM and GDP. Together, these quantities can be used to derive PEPFAR’s impacts on GDP through its impacts on ACM. Mathematically, this can be expressed as:


$$\frac{\partial GDP}{\partial PEPFAR}=\frac{\partial GDP}{\partial ACM}*\frac{\partial ACM}{\partial PEPFAR}$$

The estimation of PEPFAR’s impact on ACM $\frac{\partial ACM}{\partial PEPFAR}$ is the coefficient for the DID estimate of PEPFAR on ACM using the panel data model for the period 2004–2022; in particular, we use the time varying estimates from the heterogeneous DID panel data model. Mathematically, the ACM model with heterogeneous treatment effects is as follows:


$$ACM_{it}=\alpha_{i}+\lambda_{t}+\sum_{g\in G}\sum_{s\in S}\tau_{gs}*I\left(i\in g\right)*I\left(t=s\right)*D_{it}+\in_{it}$$

Where:

ACM_it_: ACM outcome for country i at time t.

$\alpha_{i}$: country fixed effects.

$\lambda_{t}$: time fixed effects.

$\Sigma_{g\in G}$: summation over countries in treated group.

$\Sigma_{s\in S}$: summation over time.

$\tau_{gs}$: treatment effect for group g in period s.

I(i∈g): indicator if country I belongs to treated group g.

I(t=s): indicator if time t equals s.

D_it_: binary treatment indicator (1 if treated at times t; 0 otherwise).

$\in_{it}$: error term.

This approach^17^ estimates group-time average treatment effects under staggered adoption, as well as heterogeneity in PEPFAR’s impacts on ACM over time. This helps to address potential bias that can arise in two-way fixed effects models when some members of a treatment group have not yet initiated their involvement with the PEPFAR programme. In effect, these countries remain in the comparison group until they begin participating in PEPFAR.

We tested the parallel trend assumption of the ACM DID model by regressing ACM on three variables—a dummy variable indicating countries’ PEPFAR status, a year categorical variable (with year 1990 as the reference year) and an interaction between the two. The coefficient of the interaction term indicates the difference in ACM trend between the PEPFAR and non-PEPFAR countries. A statistically insignificant coefficient of the interaction term, where its 95% CI cross 0, means that there is no statistically significant difference in ACM trend between the two groups and therefore, the parallel assumption holds between all PEPFAR and the control countries. Our tests indicate that the parallel assumption holds for all country cohorts before 2004 (see online supplemental files S1–S7 for details).

To estimate the effects of changes in ACM on GDP, $\frac{\partial GDP}{\partial ACM}$, it was necessary to correct for the potential two-way causation between ACM and GDP over time described earlier. Methods to correct for endogeneity in dynamic panel data models have been an active area of research for several decades.^21–23^ These methods recognise the difficulty of identifying instrumental variables that are correlated with an endogenous regressor (ln ACM) but uncorrelated with the dependent variable (ln GDP). Instead, these methods identify instruments by lagging the dependent variable and the endogenous regressors. The addition of dummy variables for time also helps to reduce autocorrelation problems. We seek to estimate the following equation expressing the relationship between ACM and GDP:


$$GDP_{it}=ACM_{it}\gamma +X_{it}\beta +\mu_{i}+v_{it}$$

Where:

${GDP}_{it}$: GDP for country i at time t.

${ACM}_{it}$: endogenous predictor of ${GDP}_{it}$ correlated with $\upsilon_{it}$.

γ: coefficient measuring the relationship between GDP and ACM.

$X_{it}$: a vector of observations on exogenous variables included as covariates.

β: a vector of coefficients for the exogenous covariates.

$\mu_{i}$: a vector of unobserved country effects on GDP that do not change over time.

$\nu_{it}$: residual error that varies across countries and time.

After extensive evaluation of alternative model specifications (see online supplemental files S4–S7 for details), we use GMM system estimation to estimate:


$$ln\;GDP_{it}=\alpha \;In\;GDP_{t-1}+{\overset{⌢}{ACM}}_{it}\gamma +X_{it}\beta +\mu_{i}+v_{it}$$

Where $\overset{⌢}{ACM}$_it_ is an instrumental variable based on transformations of lagged ACM that is no longer correlated with $\nu_{it}$. Specifically, we use the Stata xtabond2 procedure that implements the Blundell-Bond GMM system model for estimating dynamic panel models with endogenous regressors. This procedure estimates a system GMM model of lagged differences in ln GDP as a function of differences in instruments for ln ACM that are formed from generalised lags in ln ACM values. The model also includes time dummies to address autocorrelation, as well as country fixed effects. We find that the Hansen test fails to reject the null hypothesis that the independent variables are exogenous (see online supplemental materials for detailed results).^24^

### Findings

#### ACM reductions

Our panel data estimates indicate that PEPFAR was associated with annual ACM reductions from 2004 to 2019, with ACM for the COP and comparison group countries converging on the same value (8.4/1000) by 2020. At the end of the period, there is a rise in ACM during the COVID-19 pandemic (2020–2022; see figure 1). This rise is similar for both COP and comparison group cohorts. This represents an average annual incremental reduction of 0.25 deaths per 1000 people in the COP cohort relative to the comparison countries over 2004–2022.

**Figure 1:**
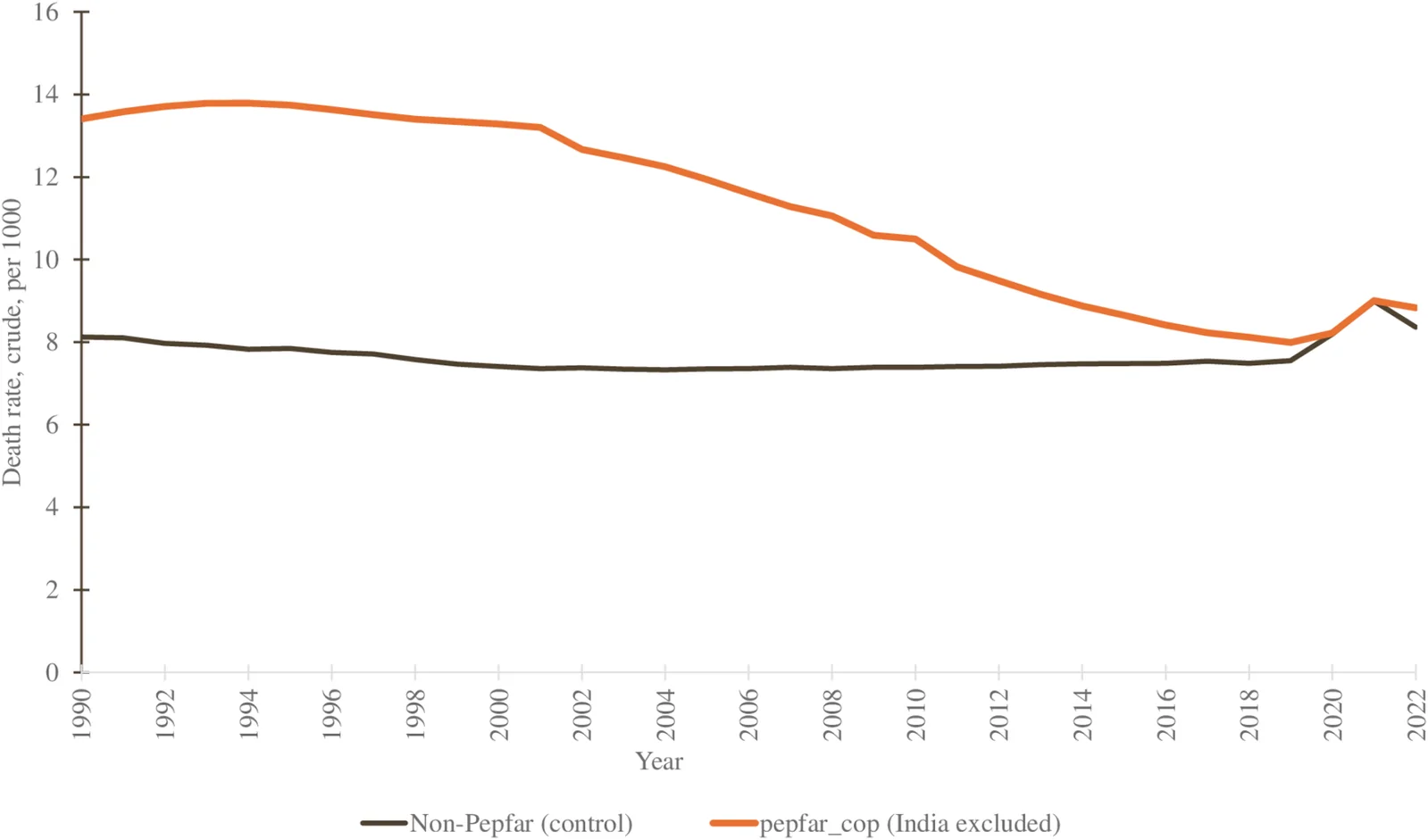
Trends in all-cause mortality rates (per 1000) for PEPFAR COP and Control Group Countries. COP, country operational plan; PEPFAR, US President’s Emergency Plan for AIDS Relief.

The estimated treatment effects represent the average ACM reduction in that year compared with 2004 relative to the corresponding change in the comparison group countries. The cumulative effects of PEPFAR on ACM relative to 2004 have increased each year, although at a diminishing rate. The results of PEPFAR’s annual impact on ACM from the heterogeneous treatment effects model are shown in figure 2.

**Figure 2:**
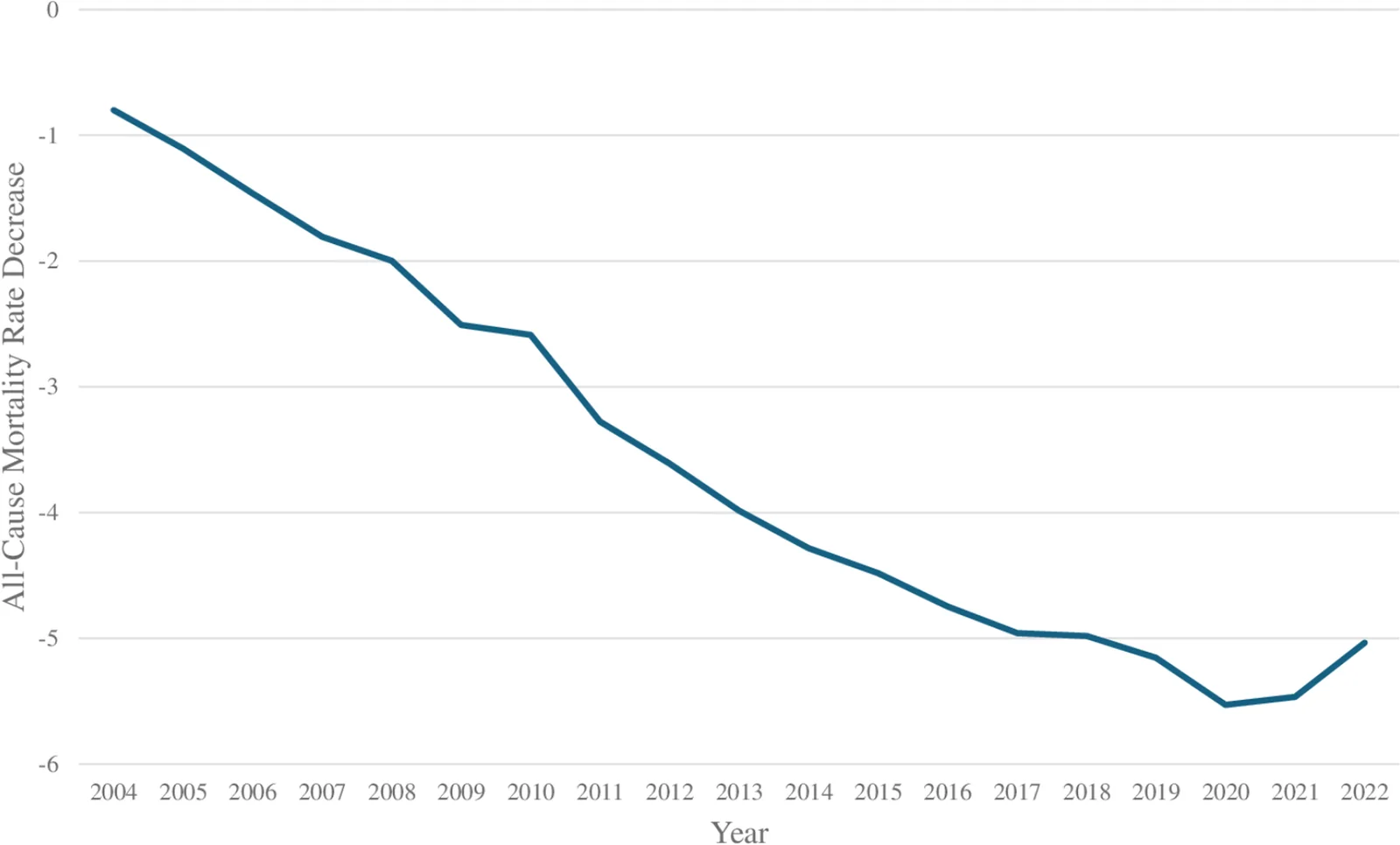
PEPFAR Impacts on ACM, 2004–2022. ACM, all-cause mortality; PEPFAR, US President’s Emergency Plan for AIDS Relief.

Table 2 reports the average ACM reduction per year in PEPFAR COP countries. In 2007, there were 1.69 fewer deaths per 1000 people when compared with what would have been expected in the absence of PEPFAR prior to 2004. That represents over 1.8 million premature deaths averted in that year alone. The number of deaths averted increases over time because the average treatment effects in the COP cohort show the number of deaths averted in that year relative to the 2003 baseline mortality rate. In 2022, this translates to 5.11 fewer deaths per 1000, indicating over 7 million deaths averted. Results indicate an average of 4.45 million deaths averted per year, or 84.58 million over the period.

**Table 2:** Treatment effect over time

Year	ATET–ACM	Incremental ACM reduction	Total population (30 COP countries)	Deaths averted (per year)
2004	−0.66	−0.66	1 011 091 363	672 086
2005	−0.99	−0.33	1 030 585 654	1 022 118
2006	−1.34	−0.35	1 050 735 533	1 407 721
2007	−1.69*	−0.35	1 071 579 699	1 807 638
2008	−1.88*	−0.20	1 092 946 775	2 057 225
2009	−2.38**	−0.50	1 114 868 262	2 657 712
2010	−2.47**	−0.09	1 137 449 476	2 814 869
2011	−3.17***	−0.69	1 160 775 447	3 676 622
2012	−3.51***	−0.34	1 184 801 644	4 156 577
2013	−3.87***	−0.36	1 209 262 927	4 681 643
2014	−4.17***	−0.30	1 234 122 570	5 150 534
2015	−4.41***	−0.23	1 259 635 580	5 549 558
2016	−4.66***	−0.25	1 285 043 031	5 986 305
2017	−4.89***	−0.23	1 310 527 279	6 410 405
2018	−4.95***	−0.05	1 336 394 456	6 609 313
2019	−5.14***	−0.19	1 362 403 077	7 003 128
2020	−5.56***	−0.42	1 388 557 379	7 716 184
2021	−5.57***	−0.01	1 414 247 752	7 878 815
2022	−5.11***	0.46	1 434 224 617	7 325 804

* p<0.05, **p<0.01, ***p<0.001.

ACM, all-cause mortality; ATET, average treatment effects on the treatment group; COP, country operational plan.

Our average estimates of PEPFAR’s impact on mortality are similar to those of 2024, Gaumer *et al*.^7^ However, our estimates more directly account for the heterogeneity of the impacts of PEPFAR over time. 2024, Gaumer *et al*^7^ report PEPFAR ACM estimates for the three periods 2004–2008, 2004–2013 and 2004–2018 which indirectly indicate that the cumulative impact of PEPFAR on ACM increased in each period. These findings are consistent with our results.

#### GDP growth

Our dynamic panel estimation data suggest that each one-point reduction in ACM is associated with a 4.9% increase in GDP in PEPFAR COP countries. Multiplying these estimates by the incremental ACM reduction (see table 3) results in an additional annual GDP growth of between 1.4% in each country. This added economic benefit cumulates to a total of 26.1% in GDP growth relative to levels that would have been expected in the absence of PEPFAR.

**Table 3:** Dynamic panel data estimation—PEPFAR impact on GDP across COP countries

	Average treatment effect	Mean ACM	% ACM reduction	PEPFAR impact on GDP	GDP—cumulative	PEPFAR impact on GDP
2003		12.47				
2004	−0.66	12.25	−5	0.26%	US$1 825 362 000 000	US$4 763 157 525
2005	−0.99	11.94	−8	0.39%	US$1 936 047 000 000	US$7 537 802 614
2006	−1.34	11.60	−11	0.53%	US$2 058 582 000 000	US$10 826 873 318
2007	−1.69	11.29	−14	0.66%	US$2 196 306 000 000	US$14 544 260 215
2008	−1.88	11.06	−15	0.74%	US$2 312 853 000 000	US$17 090 022 756
2009	−2.38	10.59	−19	0.94%	US$2 381 574 000 000	US$22 287 442 581
2010	−2.47	10.50	−20	0.97%	US$2 530 806 000 000	US$24 586 498 930
2011	−3.17	9.82	−25	1.24%	US$2 668 839 000 000	US$33 184 468 091
2012	−3.51	9.49	−28	1.38%	US$2 801 274 000 000	US$38 579 582 167
2013	−3.87	9.16	−31	1.52%	US$2 949 204 000 000	US$44 822 302 477
2014	−4.17	8.88	−33	1.64%	US$3 084 990 000 000	US$50 542 822 807
2015	−4.41	8.65	−35	1.73%	US$3 199 290 000 000	US$55 332 312 100
2016	−4.66	8.41	−37	1.83%	US$3 301 410 000 000	US$60 374 338 877
2017	−4.89	8.23	−39	1.92%	US$3 428 760 000 000	US$65 839 761 404
2018	−4.95	8.12	−40	1.94%	US$3 574 290 000 000	US$69 394 200 261
2019	−5.14	7.99	−41	2.02%	US$3 718 920 000 000	US$75 043 838 715
2020	−5.56	8.22	−45	2.18%	US$3 649 500 000 000	US$79 612 981 847
2021	−5.57	9.01	−45	2.19%	US$3 790 530 000 000	US$82 898 586 262
2022	−5.11	8.83	−41	2.01%	US$3 936 930 000 000	US$78 941 903 619
**Total**						**US$836 203 156 568**

ACM, all-cause mortality; COP, country operational plan; GDP, gross domestic product; PEPFAR, US President’s Emergency Plan for AIDS Relief.

The cumulative GDP of COP countries in 2004 was US$1.83 trillion (constant 2022 US dollars). Applying annual growth rates of 1.4% yields estimated GDP gains of US$836 billion that would not have occurred in the absence of PEPFAR across countries from 2004 to 2022.^25^ To our knowledge, these estimates are the first to directly relate PEPFAR mortality impacts with economic growth. Although our previous analysis found a positive association between PEPFAR and per-capita GDP,^5^ those estimates likely underestimated the true effects of PEPFAR on economic growth. This is because PEPFAR’s impacts on mortality reductions increase the size of the population denominator when calculating per-capita GDP. See table 3 for the average estimated impact of PEPFAR on country GDP by year, with all results reported in 2022 dollars. Results for individual countries by year are provided in online supplemental files S9–S11.

## Discussion

Current estimates of deaths averted based on reduction in ACM are more than three times higher than figures typically attributed to PEPFAR, as previous estimates have generally focused on HIV-related mortality alone.^1^ Although PEPFAR primarily supports HIV services, it also supports training healthcare workers and strengthening health systems, among other support and services. For example, it is estimated that the programme has trained 342 000 health workers, provided critical care and support for 6.6 million orphans, vulnerable children and their caregivers, and provided services to more than 2.3 million girls and young women.^1^ Many other studies have confirmed the life-saving effects of PEPFAR activities.^26–28^ While PEPFAR was associated with significant ACM reductions, further research is needed to assess whether the increase in ACM that occurred during the COVID-19 pandemic has continued—potentially driven by both its direct physical health burden and its well-documented negative mental health impacts.^29–31^ Recent funding disruptions and the dissolution of US Agency for International Development (USAID) may undermine efforts to correct these more recent ACM trends.^2^

Recent work by Gaumer *et al*^6^ using data similar to the current analysis, showed that PEPFAR resulted in significant spillover impacts on maternal and child health—lowering maternal mortality by approximately 25% and child mortality by approximately 30%. The study also found large improvements in measles, hepatitis B and diphtheria-tetanus-pertussis immunisations, as well as the prevalence of newborns protected against tetanus.^6^

The present analysis demonstrates that PEPFAR investments generate substantial positive spillover effects on GDP growth. This adds to a growing body of evidence showing that the programme has positive impacts beyond the health sector.^1 3 5–7 14 15^ Our analysis shows that, because of reduced ACM, PEPFAR-supported countries experienced higher GDP growth rates than what would have been expected without the programme. These findings show that, between 2004 and 2022, the premature deaths averted represent a total economic benefit of over $836 billion across 30 COP countries. Although these estimates are large, they are in line with expectations based on the literature. Using an international panel dataset, Rocco *et al*^25^ find that each 10% decrease in mortality rate is associated with an 8–13 percentage point increase in GDP. Our estimate that PEPFAR reduced ACM by an average of 3.5 lives per 1000 represents an approximate 41% decrease in ACM relative to baseline 2003 levels. Applying this to the estimates of Rocco *et al* yields an estimated GDP growth of 28.0–45.5%. Our dynamic panel estimation (26.1%) falls just below the range of Rocco *et al.* These findings may be seen to underscore the role of PEPFAR, not solely as a humanitarian effort, but as a strategic investment in economic stability. These findings are supported by other studies that show the widespread benefits of the programme over decades of research.^1 3 5–7 14 15 26–28^ Our finding that PEPFAR’s cumulative impacts on ACM have increased over time is consistent with the expected positive economic feedback of population health and economic growth that have been demonstrated in this literature. Improvements in population health directly impact the size and productivity of the labour force leading to economic growth. Economic growth, in turn, leads to additional investments in educational and healthcare systems resulting in further gains in economic growth.

While the present analysis demonstrates the strong association between the programme and economic growth in PEPFAR partner countries, future studies could explore the spillback effects on the US economy. The panel data indicate that household consumption comprised about 70% of GDP in PEPFAR COP countries over 2004–2022. Consequently, it is plausible that PEPFAR’s impacts on mortality directly influence household spending, among other variables. This, in turn, would be expected to have spillover effects on such factors as demand for US exports. Such effects could be estimated using similar methods to the present analysis. The direct and indirect effects of changes in the demand for US exports on US industrial production and employment could be further examined using US national accounts data and input–output analysis. Such spillback effects could be substantial. A recent analysis of spillback effects from EU external aid between 2020 and 2022 found that EU exports increased by more than the total amount of aid provided, with nearly half of every Euro spent returning to EU economies in the form of increased GDP (2025).^32^

The analysis demonstrates PEPFAR’s role in reducing ACM, along with positive economic spillover effects within partner countries. Reduced ACM plays a role in strengthening GDP, and GDP subsequently plays a strong role in future ACM reductions, reaffirming broad and interconnected benefits of the programme. While this analysis highlights substantial past health and economic benefits, future returns are at risk due to ongoing disruptions and abrupt programme reductions, the dismantling of USAID (one of the primary US Government agencies responsible for implementing PEPFAR) and continued uncertainty surrounding the programme’s funding and operations.^2^ This analysis underscores the programme’s position not only as a humanitarian effort, but as a strategic investment that bolsters economic growth and stability throughout the world.

### Limitations

The PEPFAR programme differs in the various countries in which it operates. The estimates provided are based on the average impact in these countries relative to the comparison group countries. We provide country-specific estimates of PEPFAR on ACM and GDP in the online supplemental materials; however, these estimates are generated by applying the average treatment effect estimates to the country-specific data.

Although our DID panel data models control for effects that influence health outcomes but remain constant over time, there may still be other unobserved differences between comparison group and PEPFAR countries. For example, while we controlled for other donor spending, it is still difficult to fully separate the impact of PEPFAR from other global health and development initiatives occurring in the same countries, so certain results may not be attributable to PEPFAR alone. Improvements in ACM and GDP growth almost certainly share a two-way causation over time, particularly over long periods. Although we attempted to control for this effect through our use of GMM system estimation methods and inclusion of donor spending as a covariate, there is always the question of how complete such controls are. Failure to completely control for the bidirectionality of ACM and GDP growth could introduce bias in our estimates. Nevertheless, we feel it is safe to say that the results represent ACM and related GDP outcomes in countries where PEPFAR was present versus comparison countries where it was not.

## Conclusion

This analysis contributes to the growing body of evidence demonstrating the broad economic benefits of PEPFAR in partner countries as a result of ACM reductions. The present study expands on prior work by showing how the PEPFAR programme’s effects vary over time, converting those effects into total deaths averted and quantifying how reduction in ACM influences GDP growth. When measured in terms of ACM, PEPFAR is estimated to have prevented more than 84 million deaths, substantially more than estimates based on HIV-related mortality alone. These mortality reductions translate into a total economic benefit of US$836 billion in economic benefit across countries from 2004 to 2022. Future research could explore this relationship more directly and assess the extent of potential positive spillback effects on the US economy. More broadly, these findings suggest that continued or rapid scale-downs of PEPFAR investments could result in forgoing significant future health and economic gains.

## Supplementary material

10.1136/bmjgh-2025-022885 online supplemental file 1

## Data Availability

Data are available in a public, open access repository.
