# Supplementary material for: The economic impact of deaths averted in PEPFAR countries
Source: BMJ Glob Health. 2026 Nov 11;11(11):e022885. doi: 10.1136/bmjgh-2025-022885 (PMC13756385; doi:10.1136/bmjgh-2025-022885)
Supplement: Supplementary file 1 [file bmjgh-11-11-s001.docx]

**Supplemental Materials**

**S1. Hausman test for Fixed versus Random Effects**

```
xtreg acm hiv_prevalence pepfar_pc domestic_health_pc donor_health_pc urban_pop secd_enroll fertility_rate , fe

Fixed-effects (within) regression               Number of obs     =        630
Group variable: country_code                    Number of groups  =         47

R-squared:                                      Obs per group:
     Within  = 0.5770                                         min =          2
     Between = 0.1979                                         avg =       13.4
     Overall = 0.1794                                         max =         20

                                                F(7, 576)         =     112.25
corr(u_i, Xb) = -0.6308                         Prob > F          =     0.0000

------------------------------------------------------------------------------------
               acm | Coefficient  Std. err.      t    P>|t|     [95% conf. interval]
-------------------+----------------------------------------------------------------
    hiv_prevalence |    .279257   .0719696     3.88   0.000     .1379022    .4206119
         pepfar_pc |  -.0683101   .0074821    -9.13   0.000    -.0830056   -.0536145
domestic_health_pc |   .0015786   .0001802     8.76   0.000     .0012247    .0019325
   donor_health_pc |  -.0427743   .0102265    -4.18   0.000      -.06286   -.0226886
         urban_pop |  -.0113155   .0216275    -0.52   0.601    -.0537939    .0311629
       secd_enroll |  -.0215237   .0062682    -3.43   0.001     -.033835   -.0092123
    fertility_rate |   2.249505   .1751431    12.84   0.000     1.905508    2.593502
             _cons |   2.270899   1.188613     1.91   0.057    -.0636451    4.605443
-------------------+----------------------------------------------------------------
           sigma_u |  3.9135686
           sigma_e |   .9365221
               rho |  .94583656   (fraction of variance due to u_i)
------------------------------------------------------------------------------------
F test that all u_i=0: F(46, 576) = 97.58                    Prob > F = 0.0000

estimates store fixed

xtreg acm hiv_prevalence pepfar_pc domestic_health_pc donor_health_pc urban_pop secd_enroll fertility_rate, re

Random-effects GLS regression                   Number of obs     =        630
Group variable: country_code                    Number of groups  =         47

R-squared:                                      Obs per group:
     Within  = 0.5701                                         min =          2
     Between = 0.2527                                         avg =       13.4
     Overall = 0.2250                                         max =         20

                                                Wald chi2(7)      =     713.99
corr(u_i, X) = 0 (assumed)                      Prob > chi2       =     0.0000

------------------------------------------------------------------------------------
               acm | Coefficient  Std. err.      z    P>|z|     [95% conf. interval]
-------------------+----------------------------------------------------------------
    hiv_prevalence |   .3153306   .0455685     6.92   0.000     .2260179    .4046433
         pepfar_pc |  -.0703856   .0076611    -9.19   0.000    -.0854011     -.05537
domestic_health_pc |   .0016319   .0001835     8.89   0.000     .0012722    .0019917
   donor_health_pc |  -.0541647   .0102487    -5.29   0.000    -.0742518   -.0340775
         urban_pop |   .0027495   .0157057     0.18   0.861    -.0280332    .0335322
       secd_enroll |  -.0253133   .0059533    -4.25   0.000    -.0369816    -.013645
    fertility_rate |     1.7604   .1583062    11.12   0.000     1.450126    2.070675
             _cons |   3.034724   1.076055     2.82   0.005     .9256942    5.143754
-------------------+----------------------------------------------------------------
           sigma_u |  2.4881361
           sigma_e |   .9365221
               rho |  .87590737   (fraction of variance due to u_i)
------------------------------------------------------------------------------------

estimates store random

hausman fixed random, sigmamore

                 ---- Coefficients ----
             |      (b)          (B)            (b-B)     sqrt(diag(V_b-V_B))
             |     fixed        random       Difference       Std. err.
-------------+----------------------------------------------------------------
hiv_preval~e |     .279257     .3153306       -.0360736        .0592447
   pepfar_pc |   -.0683101    -.0703856        .0020755        .0012983
domestic_h~c |    .0015786     .0016319       -.0000533        .0000364
donor_heal~c |   -.0427743    -.0541647        .0113904        .0027853
   urban_pop |   -.0113155     .0027495        -.014065        .0160567
 secd_enroll |   -.0215237    -.0253133        .0037896        .0026334
fertility_~e |    2.249505       1.7604        .4891043        .0895738
------------------------------------------------------------------------------
                          b = Consistent under H0 and Ha; obtained from xtreg.
           B = Inconsistent under Ha, efficient under H0; obtained from xtreg.

Test of H0: Difference in coefficients not systematic

    chi2(7) = (b-B)'[(V_b-V_B)^(-1)](b-B)
            =  51.72
Prob > chi2 = 0.0000
```

**S2. ACM DID Model Using Heterogeneous Treatment Effects**

```
xthdidregress twfe (acm) (treated), group(country_code) vce(robust)
note: variable _did_cohort, containing cohort indicators formed by treatment variable treated and group variable country_code, was added to the dataset.

Treatment and time information

Time variable: year
Time interval: 1990 to 2022
Control:       _did_cohort = 0
Treatment:     _did_cohort > 0
-------------------------------
                  | _did_cohort
------------------+------------
Number of cohorts |           2
------------------+------------
Number of obs     |
    Never treated |        1950
             2004 |         990
-------------------------------

Heterogeneous-treatment-effects regression            Number of obs    = 2,940
                                                      Number of panels =    91
Estimator:       Two-way fixed effects
Panel variable:  country_code
Treatment level: country_code
Control group:   Never treated
Heterogeneity:   Cohort and time

                          (Std. err. adjusted for 91 clusters in country_code)
------------------------------------------------------------------------------
             |               Robust
Cohort       |       ATET   std. err.      t    P>|t|     [95% conf. interval]
-------------+----------------------------------------------------------------
        year |
       2004  |   -.664713   .7115718    -0.93   0.353    -2.078375    .7489486
       2005  |   -.991784   .7671413    -1.29   0.199    -2.515844     .532276
       2006  |  -1.339748   .7923615    -1.69   0.094    -2.913912    .2344165
       2007  |  -1.686891   .8152662    -2.07   0.041     -3.30656   -.0672225
       2008  |  -1.882274    .841907    -2.24   0.028    -3.554869   -.2096785
       2009  |   -2.38388   .8386879    -2.84   0.006     -4.05008   -.7176804
       2010  |   -2.47472   .8766366    -2.82   0.006    -4.216311   -.7331278
       2011  |  -3.167384   .8206497    -3.86   0.000    -4.797748    -1.53702
       2012  |  -3.508247    .826603    -4.24   0.000    -5.150438   -1.866056
       2013  |  -3.871485   .8409942    -4.60   0.000    -5.542266   -2.200703
       2014  |  -4.173438    .852027    -4.90   0.000    -5.866138   -2.480738
       2015  |  -4.405685   .8565463    -5.14   0.000    -6.107364   -2.704006
       2016  |  -4.658447   .8657059    -5.38   0.000    -6.378323   -2.938571
       2017  |   -4.89147   .8767466    -5.58   0.000     -6.63328    -3.14966
       2018  |   -4.94563   .8838918    -5.60   0.000    -6.701636   -3.189625
       2019  |  -5.140276   .8956358    -5.74   0.000    -6.919613    -3.36094
       2020  |  -5.556979   .9366064    -5.93   0.000    -7.417711   -3.696247
       2021  |  -5.571029   1.051714    -5.30   0.000    -7.660442   -3.481615
       2022  |   -5.10785   1.077119    -4.74   0.000    -7.247735   -2.967964
------------------------------------------------------------------------------

estat aggregation, cohort

ATET over cohort                                         Number of obs = 2,940

                          (Std. err. adjusted for 91 clusters in country_code)
------------------------------------------------------------------------------
             |               Robust
      Cohort |       ATET   std. err.      t    P>|t|     [95% conf. interval]
-------------+----------------------------------------------------------------
        2004 |  -3.495891   .8319387    -4.20   0.000    -5.148683     -1.8431
------------------------------------------------------------------------------

estat aggregation, time

ATET over time                                           Number of obs = 2,940

                          (Std. err. adjusted for 91 clusters in country_code)
------------------------------------------------------------------------------
             |               Robust
        Time |       ATET   std. err.      t    P>|t|     [95% conf. interval]
-------------+----------------------------------------------------------------
        2004 |   -.664713   .7115718    -0.93   0.353    -2.078375    .7489486
        2005 |   -.991784   .7671413    -1.29   0.199    -2.515844     .532276
        2006 |  -1.339748   .7923615    -1.69   0.094    -2.913912    .2344165
        2007 |  -1.686891   .8152662    -2.07   0.041     -3.30656   -.0672225
        2008 |  -1.882274    .841907    -2.24   0.028    -3.554869   -.2096785
        2009 |   -2.38388   .8386879    -2.84   0.006     -4.05008   -.7176804
        2010 |   -2.47472   .8766366    -2.82   0.006    -4.216311   -.7331278
        2011 |  -3.167384   .8206497    -3.86   0.000    -4.797748    -1.53702
        2012 |  -3.508247    .826603    -4.24   0.000    -5.150438   -1.866056
        2013 |  -3.871485   .8409942    -4.60   0.000    -5.542266   -2.200703
        2014 |  -4.173438    .852027    -4.90   0.000    -5.866138   -2.480738
        2015 |  -4.405685   .8565463    -5.14   0.000    -6.107364   -2.704006
        2016 |  -4.658447   .8657059    -5.38   0.000    -6.378323   -2.938571
        2017 |   -4.89147   .8767466    -5.58   0.000     -6.63328    -3.14966
        2018 |   -4.94563   .8838918    -5.60   0.000    -6.701636   -3.189625
        2019 |  -5.140276   .8956358    -5.74   0.000    -6.919613    -3.36094
        2020 |  -5.556979   .9366064    -5.93   0.000    -7.417711   -3.696247
        2021 |  -5.571029   1.051714    -5.30   0.000    -7.660442   -3.481615
        2022 |   -5.10785   1.077119    -4.74   0.000    -7.247735   -2.967964
------------------------------------------------------------------------------
```

**S3. ACM Common DID Model**

```
xtdidregress (acm) (treated), group(country_code) time(year) vce(robust)

Treatment and time information

Time variable: year
Control:       treated = 0
Treatment:     treated = 1
-----------------------------------
             |   Control  Treatment
-------------+---------------------
Group        |
country_code |        61         30
-------------+---------------------
Time         |
     Minimum |      1990       2004
     Maximum |      2002       2004
-----------------------------------

Difference-in-differences regression                     Number of obs = 2,940
Data type: Longitudinal

                          (Std. err. adjusted for 91 clusters in country_code)
------------------------------------------------------------------------------
             |               Robust
         acm | Coefficient  std. err.      t    P>|t|     [95% conf. interval]
-------------+----------------------------------------------------------------
ATET         |
     treated |
   (1 vs 0)  |  -3.495891   .8284962    -4.22   0.000    -5.141843   -1.849939
------------------------------------------------------------------------------
Note: ATET estimate adjusted for panel effects and time effects.
Note: Treatment occurs at different times.
```

**S4. Simple Panel Data Model of GNP and Endogeneity-Adjusted Version**

```
*Simple Panel Data Model of GNP
. xtreg ln_gdp acm urban_pop secd_enroll i.year, fe

Fixed-effects (within) regression               Number of obs     =      2,147
Group variable: country_code                    Number of groups  =         87

R-squared:                                      Obs per group:
     Within  = 0.7847                                         min =          1
     Between = 0.0629                                         avg =       24.7
     Overall = 0.1463                                         max =         33

                                                F(35, 2025)       =     210.84
corr(u_i, Xb) = 0.1182                          Prob > F          =     0.0000

------------------------------------------------------------------------------
      ln_gdp | Coefficient  Std. err.      t    P>|t|     [95% conf. interval]
-------------+----------------------------------------------------------------
         acm |  -.0234958   .0019298   -12.18   0.000    -.0272804   -.0197111
   urban_pop |   .0172704   .0014591    11.84   0.000     .0144089    .0201319
 secd_enroll |   .0019084   .0003871     4.93   0.000     .0011493    .0026675
             |
        year |
       1991  |  -.0108439   .0308336    -0.35   0.725    -.0713128     .049625
       1992  |   .0045591    .030652     0.15   0.882    -.0555538    .0646719
       1993  |   .0117576   .0308707     0.38   0.703    -.0487841    .0722992
       1994  |   .0150508   .0307015     0.49   0.624     -.045159    .0752607
       1995  |   .0448506   .0306681     1.46   0.144    -.0152936    .1049949
       1996  |   .0974727   .0305351     3.19   0.001     .0375893    .1573561
       1997  |   .1390758   .0305914     4.55   0.000      .079082    .1990697
       1998  |   .1694885   .0307341     5.51   0.000     .1092147    .2297622
       1999  |   .1873181   .0308224     6.08   0.000     .1268712    .2477649
       2000  |    .208854   .0310131     6.73   0.000     .1480331     .269675
       2001  |   .2257149   .0310691     7.26   0.000     .1647841    .2866456
       2002  |   .2558373    .032933     7.77   0.000     .1912512    .3204235
       2003  |   .2747853     .03378     8.13   0.000     .2085381    .3410325
       2004  |   .3269626    .033274     9.83   0.000     .2617077    .3922175
       2005  |   .3881506    .033538    11.57   0.000      .322378    .4539231
       2006  |   .4045896   .0344575    11.74   0.000     .3370138    .4721655
       2007  |   .4558387   .0334064    13.65   0.000     .3903242    .5213532
       2008  |   .5016484   .0344827    14.55   0.000     .4340231    .5692736
       2009  |   .4947462    .034606    14.30   0.000      .426879    .5626134
       2010  |   .5399064   .0345272    15.64   0.000     .4721939     .607619
       2011  |   .5635612   .0350345    16.09   0.000     .4948539    .6322686
       2012  |   .5741888   .0351851    16.32   0.000     .5051861    .6431915
       2013  |   .5955251   .0361839    16.46   0.000     .5245636    .6664867
       2014  |   .6480119   .0358836    18.06   0.000     .5776393    .7183846
       2015  |   .6500439    .036175    17.97   0.000     .5790997    .7209881
       2016  |   .6886146   .0365526    18.84   0.000     .6169299    .7602993
       2017  |   .7419807   .0381501    19.45   0.000     .6671632    .8167982
       2018  |   .7760721   .0379624    20.44   0.000     .7016226    .8505215
       2019  |   .7701967    .038162    20.18   0.000      .695356    .8450375
       2020  |   .6885701   .0379113    18.16   0.000     .6142208    .7629193
       2021  |   .7725027   .0373794    20.67   0.000     .6991967    .8458088
       2022  |   .7902107   .0423573    18.66   0.000     .7071423     .873279
             |
       _cons |   22.54778    .072305   311.84   0.000     22.40598    22.68958
-------------+----------------------------------------------------------------
     sigma_u |  2.2034518
     sigma_e |  .18937788
         rho |  .99266745   (fraction of variance due to u_i)
------------------------------------------------------------------------------
F test that all u_i=0: F(86, 2025) = 2477.55                 Prob > F = 0.0000

*ln_ACM endogenous with covariates including time dummies
xtabond2 ln_gdp L.ln_gdp ln_acm urban_pop secd_enroll i.year, gmm(L.ln_gdp ln_acm,
> lag(2 3)) twostep robust small
Favoring space over speed. To switch, type or click on mata: mata set matafavor speed
> , perm.
1990b.year dropped due to collinearity
1999.year dropped due to collinearity
Warning: Number of instruments may be large relative to number of observations.
Warning: Two-step estimated covariance matrix of moments is singular.
  Using a generalized inverse to calculate optimal weighting matrix for two-step esti
> mation.
  Difference-in-Sargan/Hansen statistics may be negative.

Dynamic panel-data estimation, two-step system GMM
------------------------------------------------------------------------------
Group variable: country_code                    Number of obs      =      2068
Time variable : year                            Number of groups   =        87
Number of instruments = 182                     Obs per group: min =         1
F(35, 86)     =  3.29e+06                                      avg =     23.77
Prob > F      =     0.000                                      max =        32
------------------------------------------------------------------------------
             |              Corrected
      ln_gdp | Coefficient  std. err.      t    P>|t|     [95% conf. interval]
-------------+----------------------------------------------------------------
      ln_gdp |
         L1. |    1.00428   .0054295   184.97   0.000     .9934863    1.015073
             |
      ln_acm |  -.0489411   .0212824    -2.30   0.024    -.0912491   -.0066331
   urban_pop |   .0010992   .0007456     1.47   0.144    -.0003829    .0025814
 secd_enroll |  -.0012048   .0006491    -1.86   0.067    -.0024951    .0000854
             |
        year |
       1991  |  -.0359615   .0135875    -2.65   0.010    -.0629726   -.0089504
       1992  |  -.0078791   .0102888    -0.77   0.446    -.0283324    .0125743
       1993  |  -.0156715   .0094584    -1.66   0.101    -.0344743    .0031312
       1994  |   -.011617   .0141413    -0.82   0.414     -.039729    .0164949
       1995  |   .0107183   .0079903     1.34   0.183    -.0051658    .0266024
       1996  |   .0244785   .0062527     3.91   0.000     .0120486    .0369084
       1997  |   .0190866    .007889     2.42   0.018     .0034038    .0347695
       1998  |   .0040596   .0070351     0.58   0.565    -.0099257    .0180449
       2000  |   .0054548   .0059183     0.92   0.359    -.0063104      .01722
       2001  |  -.0021407    .006859    -0.31   0.756    -.0157759    .0114944
       2002  |  -.0039857    .007976    -0.50   0.619    -.0198414      .01187
       2003  |   .0128382   .0083332     1.54   0.127    -.0037277    .0294041
       2004  |   .0327503   .0085901     3.81   0.000     .0156738    .0498268
       2005  |   .0287075   .0080942     3.55   0.001     .0126167    .0447983
       2006  |   .0337484   .0082912     4.07   0.000      .017266    .0502308
       2007  |   .0332134   .0086121     3.86   0.000     .0160932    .0503337
       2008  |   .0226938   .0084783     2.68   0.009     .0058395    .0395481
       2009  |  -.0175079   .0115772    -1.51   0.134    -.0405226    .0055068
       2010  |   .0270597   .0106305     2.55   0.013     .0059269    .0481925
       2011  |   .0227356   .0095082     2.39   0.019     .0038339    .0416373
       2012  |   .0049355   .0106943     0.46   0.646     -.016324    .0261951
       2013  |   .0097227    .011045     0.88   0.381     -.012234    .0316794
       2014  |   .0158008   .0106589     1.48   0.142    -.0053884      .03699
       2015  |   .0135647    .011122     1.22   0.226    -.0085451    .0356744
       2016  |   .0109805   .0108615     1.01   0.315    -.0106114    .0325724
       2017  |   .0166343   .0109459     1.52   0.132    -.0051254    .0383941
       2018  |   .0171399    .011196     1.53   0.129     -.005117    .0393968
       2019  |   .0140568   .0116338     1.21   0.230    -.0090704    .0371841
       2020  |  -.0627071    .015262    -4.11   0.000    -.0930469   -.0323673
       2021  |   .0345099   .0125432     2.75   0.007     .0095748     .059445
       2022  |   .0289919    .013751     2.11   0.038     .0016558    .0563279
             |
       _cons |   .0555307   .1153292     0.48   0.631    -.1737362    .2847976
------------------------------------------------------------------------------
Instruments for first differences equation
  GMM-type (missing=0, separate instruments for each period unless collapsed)
    L(2/3).(L.ln_gdp ln_acm)
Instruments for levels equation
  Standard
    _cons
  GMM-type (missing=0, separate instruments for each period unless collapsed)
    DL.(L.ln_gdp ln_acm)
------------------------------------------------------------------------------
Arellano-Bond test for AR(1) in first differences: z =  -3.07  Pr > z =  0.002
Arellano-Bond test for AR(2) in first differences: z =  -1.67  Pr > z =  0.094
------------------------------------------------------------------------------
Sargan test of overid. restrictions: chi2(146)  = 433.73  Prob > chi2 =  0.000
  (Not robust, but not weakened by many instruments.)
Hansen test of overid. restrictions: chi2(146)  =  55.25  Prob > chi2 =  1.000
  (Robust, but weakened by many instruments.)

Difference-in-Hansen tests of exogeneity of instrument subsets:
  GMM instruments for levels
    Hansen test excluding group:     chi2(85)   =  48.47  Prob > chi2 =  1.000
    Difference (null H = exogenous): chi2(61)   =   6.78  Prob > chi2 =  1.000
```

**S5. Model Results Including India**

```
. xthdidregress twfe (acm) (treated), group(country_code) vce(robust)
note: variable _did_cohort, containing cohort indicators formed by treatment
      variable treated and group variable country_code, was added to the
      dataset using the estimation sample.

Treatment and time information

Time variable: year
Time interval: 1990 to 2022
Control:       _did_cohort = 0
Treatment:     _did_cohort > 0
-------------------------------
                  | _did_cohort
------------------+------------
Number of cohorts |           2
------------------+------------
Number of obs     |
    Never treated |        1950
             2004 |        1023
-------------------------------

Heterogeneous-treatment-effects regression            Number of obs    = 2,973
                                                      Number of panels =    92
Estimator:       Two-way fixed effects
Panel variable:  country_code
Treatment level: country_code
Control group:   Never treated
Heterogeneity:   Cohort and time
Over:            Cohort

                          (Std. err. adjusted for 92 clusters in country_code)
------------------------------------------------------------------------------
             |               Robust
Cohort       |       ATET   std. err.      t    P>|t|     [95% conf. interval]
-------------+----------------------------------------------------------------
        year |
       2004  |  -.6709299   .6893563    -0.97   0.333    -2.040251    .6983918
       2005  |  -.9934095   .7431005    -1.34   0.185    -2.469487    .4826683
       2006  |  -1.334767   .7675431    -1.74   0.085    -2.859397    .1898632
       2007  |  -1.674983   .7899119    -2.12   0.037    -3.244046   -.1059202
       2008  |  -1.865742   .8157687    -2.29   0.025    -3.486166   -.2453178
       2009  |  -2.354639   .8131751    -2.90   0.005    -3.969912   -.7393665
       2010  |   -2.44573   .8498489    -2.88   0.005     -4.13385   -.7576094
       2011  |  -3.120313   .7968804    -3.92   0.000    -4.703218   -1.537408
       2012  |  -3.455187   .8032554    -4.30   0.000    -5.050755   -1.859619
       2013  |  -3.813054   .8179234    -4.66   0.000    -5.437759    -2.18835
       2014  |  -4.111357   .8289475    -4.96   0.000    -5.757959   -2.464755
       2015  |  -4.340215   .8334613    -5.21   0.000    -5.995783   -2.684646
       2016  |  -4.587284   .8428638    -5.44   0.000     -6.26153   -2.913039
       2017  |  -4.814714   .8541715    -5.64   0.000     -6.51142   -3.118007
       2018  |  -4.863784    .861387    -5.65   0.000    -6.574823   -3.152744
       2019  |  -5.052159   .8736162    -5.78   0.000     -6.78749   -3.316828
       2020  |  -5.455576   .9150356    -5.96   0.000    -7.273181    -3.63797
       2021  |  -5.427269   1.032479    -5.26   0.000    -7.478162   -3.376377
       2022  |  -4.970604   1.054868    -4.71   0.000     -7.06597   -2.875239
------------------------------------------------------------------------------

.
. estat aggregation, cohort

ATET over cohort                                         Number of obs = 2,973

                          (Std. err. adjusted for 92 clusters in country_code)
------------------------------------------------------------------------------
             |               Robust
      Cohort |       ATET   std. err.      t    P>|t|     [95% conf. interval]
-------------+----------------------------------------------------------------
        2004 |  -3.439564   .8086362    -4.25   0.000     -5.04582   -1.833307
------------------------------------------------------------------------------

.
. estat aggregation, time

ATET over time                                           Number of obs = 2,973

                          (Std. err. adjusted for 92 clusters in country_code)
------------------------------------------------------------------------------
             |               Robust
        Time |       ATET   std. err.      t    P>|t|     [95% conf. interval]
-------------+----------------------------------------------------------------
        2004 |  -.6709299   .6893563    -0.97   0.333    -2.040251    .6983918
        2005 |  -.9934095   .7431005    -1.34   0.185    -2.469487    .4826683
        2006 |  -1.334767   .7675431    -1.74   0.085    -2.859397    .1898632
        2007 |  -1.674983   .7899119    -2.12   0.037    -3.244046   -.1059202
        2008 |  -1.865742   .8157687    -2.29   0.025    -3.486166   -.2453178
        2009 |  -2.354639   .8131751    -2.90   0.005    -3.969912   -.7393665
        2010 |   -2.44573   .8498489    -2.88   0.005     -4.13385   -.7576094
        2011 |  -3.120313   .7968804    -3.92   0.000    -4.703218   -1.537408
        2012 |  -3.455187   .8032554    -4.30   0.000    -5.050755   -1.859619
        2013 |  -3.813054   .8179234    -4.66   0.000    -5.437759    -2.18835
        2014 |  -4.111357   .8289475    -4.96   0.000    -5.757959   -2.464755
        2015 |  -4.340215   .8334613    -5.21   0.000    -5.995783   -2.684646
        2016 |  -4.587284   .8428638    -5.44   0.000     -6.26153   -2.913039
        2017 |  -4.814714   .8541715    -5.64   0.000     -6.51142   -3.118007
        2018 |  -4.863784    .861387    -5.65   0.000    -6.574823   -3.152744
        2019 |  -5.052159   .8736162    -5.78   0.000     -6.78749   -3.316828
        2020 |  -5.455576   .9150356    -5.96   0.000    -7.273181    -3.63797
        2021 |  -5.427269   1.032479    -5.26   0.000    -7.478162   -3.376377
        2022 |  -4.970604   1.054868    -4.71   0.000     -7.06597   -2.875239
------------------------------------------------------------------------------

.
. estat aggregation, dynamic graph

Duration of exposure ATET                                Number of obs = 2,973

                          (Std. err. adjusted for 92 clusters in country_code)
------------------------------------------------------------------------------
             |               Robust
    Exposure |       ATET   std. err.      t    P>|t|     [95% conf. interval]
-------------+----------------------------------------------------------------
           0 |  -.6709299   .6893563    -0.97   0.333    -2.040251    .6983918
           1 |  -.9934095   .7431005    -1.34   0.185    -2.469487    .4826683
           2 |  -1.334767   .7675431    -1.74   0.085    -2.859397    .1898632
           3 |  -1.674983   .7899119    -2.12   0.037    -3.244046   -.1059202
           4 |  -1.865742   .8157687    -2.29   0.025    -3.486166   -.2453178
           5 |  -2.354639   .8131751    -2.90   0.005    -3.969912   -.7393665
           6 |   -2.44573   .8498489    -2.88   0.005     -4.13385   -.7576094
           7 |  -3.120313   .7968804    -3.92   0.000    -4.703218   -1.537408
           8 |  -3.455187   .8032554    -4.30   0.000    -5.050755   -1.859619
           9 |  -3.813054   .8179234    -4.66   0.000    -5.437759    -2.18835
          10 |  -4.111357   .8289475    -4.96   0.000    -5.757959   -2.464755
          11 |  -4.340215   .8334613    -5.21   0.000    -5.995783   -2.684646
          12 |  -4.587284   .8428638    -5.44   0.000     -6.26153   -2.913039
          13 |  -4.814714   .8541715    -5.64   0.000     -6.51142   -3.118007
          14 |  -4.863784    .861387    -5.65   0.000    -6.574823   -3.152744
          15 |  -5.052159   .8736162    -5.78   0.000     -6.78749   -3.316828
          16 |  -5.455576   .9150356    -5.96   0.000    -7.273181    -3.63797
          17 |  -5.427269   1.032479    -5.26   0.000    -7.478162   -3.376377
          18 |  -4.970604   1.054868    -4.71   0.000     -7.06597   -2.875239
------------------------------------------------------------------------------
Note: Exposure is the number of periods since the first treatment time.

.
. *common panel data DID treatment effects model for ACM

.
. xtdidregress (acm) (treated), group(country_code) time(year) vce(robust)

Treatment and time information

Time variable: year
Control:       treated = 0
Treatment:     treated = 1
-----------------------------------
             |   Control  Treatment
-------------+---------------------
Group        |
country_code |        61         31
-------------+---------------------
Time         |
     Minimum |      1990       2004
     Maximum |      2002       2004
-----------------------------------

Difference-in-differences regression                     Number of obs = 2,973
Data type: Longitudinal

                          (Std. err. adjusted for 92 clusters in country_code)
------------------------------------------------------------------------------
             |               Robust
         acm | Coefficient  std. err.      t    P>|t|     [95% conf. interval]
-------------+----------------------------------------------------------------
ATET         |
     treated |
   (1 vs 0)  |  -3.439564   .8053278    -4.27   0.000    -5.039249   -1.839879
------------------------------------------------------------------------------
Note: ATET estimate adjusted for panel effects and time effects.
Note: Treatment occurs at different times.

.
.
. *heterogeneous DID treatment effects model for ln_ACM using FE

.
. xthdidregress twfe (ln_acm) (treated), group(country_code) vce(robust)
note: variable _did_cohort, containing cohort indicators formed by treatment
      variable treated and group variable country_code, was added to the
      dataset using the estimation sample.

Treatment and time information

Time variable: year
Time interval: 1990 to 2022
Control:       _did_cohort = 0
Treatment:     _did_cohort > 0
-------------------------------
                  | _did_cohort
------------------+------------
Number of cohorts |           2
------------------+------------
Number of obs     |
    Never treated |        1950
             2004 |        1023
-------------------------------

Heterogeneous-treatment-effects regression            Number of obs    = 2,973
                                                      Number of panels =    92
Estimator:       Two-way fixed effects
Panel variable:  country_code
Treatment level: country_code
Control group:   Never treated
Heterogeneity:   Cohort and time
Over:            Cohort

                          (Std. err. adjusted for 92 clusters in country_code)
------------------------------------------------------------------------------
             |               Robust
Cohort       |       ATET   std. err.      t    P>|t|     [95% conf. interval]
-------------+----------------------------------------------------------------
        year |
       2004  |  -.0026597   .0467695    -0.06   0.955    -.0955617    .0902423
       2005  |  -.0300358   .0506583    -0.59   0.555    -.1306623    .0705908
       2006  |  -.0611153   .0529083    -1.16   0.251    -.1662111    .0439806
       2007  |  -.0898356   .0554774    -1.62   0.109    -.2000346    .0203634
       2008  |  -.1054502   .0587022    -1.80   0.076    -.2220549    .0111545
       2009  |  -.1495083   .0594842    -2.51   0.014    -.2676664   -.0313502
       2010  |  -.1557052    .063309    -2.46   0.016    -.2814607   -.0299497
       2011  |   -.213523   .0608289    -3.51   0.001    -.3343522   -.0926939
       2012  |  -.2489106   .0630832    -3.95   0.000    -.3742176   -.1236036
       2013  |  -.2855963   .0660357    -4.32   0.000    -.4167681   -.1544246
       2014  |  -.3202007   .0679022    -4.72   0.000    -.4550802   -.1853213
       2015  |   -.343556   .0687096    -5.00   0.000    -.4800392   -.2070727
       2016  |  -.3735541   .0707023    -5.28   0.000    -.5139955   -.2331127
       2017  |  -.3973228   .0724397    -5.48   0.000    -.5412153   -.2534303
       2018  |  -.4051442   .0730387    -5.55   0.000    -.5502266   -.2600618
       2019  |  -.4285145   .0749446    -5.72   0.000    -.5773827   -.2796463
       2020  |  -.4816192   .0779636    -6.18   0.000    -.6364842   -.3267542
       2021  |  -.4710236    .088598    -5.32   0.000    -.6470127   -.2950345
       2022  |  -.4374314   .0910316    -4.81   0.000    -.6182544   -.2566084
------------------------------------------------------------------------------

.
. estat aggregation, cohort

ATET over cohort                                         Number of obs = 2,973

                          (Std. err. adjusted for 92 clusters in country_code)
------------------------------------------------------------------------------
             |               Robust
      Cohort |       ATET   std. err.      t    P>|t|     [95% conf. interval]
-------------+----------------------------------------------------------------
        2004 |  -.2631951    .062963    -4.18   0.000    -.3882634   -.1381267
------------------------------------------------------------------------------

.
. estat aggregation, time

ATET over time                                           Number of obs = 2,973

                          (Std. err. adjusted for 92 clusters in country_code)
------------------------------------------------------------------------------
             |               Robust
        Time |       ATET   std. err.      t    P>|t|     [95% conf. interval]
-------------+----------------------------------------------------------------
        2004 |  -.0026597   .0467695    -0.06   0.955    -.0955617    .0902423
        2005 |  -.0300358   .0506583    -0.59   0.555    -.1306623    .0705908
        2006 |  -.0611153   .0529083    -1.16   0.251    -.1662111    .0439806
        2007 |  -.0898356   .0554774    -1.62   0.109    -.2000346    .0203634
        2008 |  -.1054502   .0587022    -1.80   0.076    -.2220549    .0111545
        2009 |  -.1495083   .0594842    -2.51   0.014    -.2676664   -.0313502
        2010 |  -.1557052    .063309    -2.46   0.016    -.2814607   -.0299497
        2011 |   -.213523   .0608289    -3.51   0.001    -.3343522   -.0926939
        2012 |  -.2489106   .0630832    -3.95   0.000    -.3742176   -.1236036
        2013 |  -.2855963   .0660357    -4.32   0.000    -.4167681   -.1544246
        2014 |  -.3202007   .0679022    -4.72   0.000    -.4550802   -.1853213
        2015 |   -.343556   .0687096    -5.00   0.000    -.4800392   -.2070727
        2016 |  -.3735541   .0707023    -5.28   0.000    -.5139955   -.2331127
        2017 |  -.3973228   .0724397    -5.48   0.000    -.5412153   -.2534303
        2018 |  -.4051442   .0730387    -5.55   0.000    -.5502266   -.2600618
        2019 |  -.4285145   .0749446    -5.72   0.000    -.5773827   -.2796463
        2020 |  -.4816192   .0779636    -6.18   0.000    -.6364842   -.3267542
        2021 |  -.4710236    .088598    -5.32   0.000    -.6470127   -.2950345
        2022 |  -.4374314   .0910316    -4.81   0.000    -.6182544   -.2566084
------------------------------------------------------------------------------

.
. estat aggregation, dynamic graph

Duration of exposure ATET                                Number of obs = 2,973

                          (Std. err. adjusted for 92 clusters in country_code)
------------------------------------------------------------------------------
             |               Robust
    Exposure |       ATET   std. err.      t    P>|t|     [95% conf. interval]
-------------+----------------------------------------------------------------
           0 |  -.0026597   .0467695    -0.06   0.955    -.0955617    .0902423
           1 |  -.0300358   .0506583    -0.59   0.555    -.1306623    .0705908
           2 |  -.0611153   .0529083    -1.16   0.251    -.1662111    .0439806
           3 |  -.0898356   .0554774    -1.62   0.109    -.2000346    .0203634
           4 |  -.1054502   .0587022    -1.80   0.076    -.2220549    .0111545
           5 |  -.1495083   .0594842    -2.51   0.014    -.2676664   -.0313502
           6 |  -.1557052    .063309    -2.46   0.016    -.2814607   -.0299497
           7 |   -.213523   .0608289    -3.51   0.001    -.3343522   -.0926939
           8 |  -.2489106   .0630832    -3.95   0.000    -.3742176   -.1236036
           9 |  -.2855963   .0660357    -4.32   0.000    -.4167681   -.1544246
          10 |  -.3202007   .0679022    -4.72   0.000    -.4550802   -.1853213
          11 |   -.343556   .0687096    -5.00   0.000    -.4800392   -.2070727
          12 |  -.3735541   .0707023    -5.28   0.000    -.5139955   -.2331127
          13 |  -.3973228   .0724397    -5.48   0.000    -.5412153   -.2534303
          14 |  -.4051442   .0730387    -5.55   0.000    -.5502266   -.2600618
          15 |  -.4285145   .0749446    -5.72   0.000    -.5773827   -.2796463
          16 |  -.4816192   .0779636    -6.18   0.000    -.6364842   -.3267542
          17 |  -.4710236    .088598    -5.32   0.000    -.6470127   -.2950345
          18 |  -.4374314   .0910316    -4.81   0.000    -.6182544   -.2566084
------------------------------------------------------------------------------
Note: Exposure is the number of periods since the first treatment time.

.
. *common panel data DID treatment effects model for ACM

.
. xtdidregress (ln_acm) (treated), group(country_code) time(year) vce(robust)

Treatment and time information

Time variable: year
Control:       treated = 0
Treatment:     treated = 1
-----------------------------------
             |   Control  Treatment
-------------+---------------------
Group        |
country_code |        61         31
-------------+---------------------
Time         |
     Minimum |      1990       2004
     Maximum |      2002       2004
-----------------------------------

Difference-in-differences regression                     Number of obs = 2,973
Data type: Longitudinal

                          (Std. err. adjusted for 92 clusters in country_code)
------------------------------------------------------------------------------
             |               Robust
      ln_acm | Coefficient  std. err.      t    P>|t|     [95% conf. interval]
-------------+----------------------------------------------------------------
ATET         |
     treated |
   (1 vs 0)  |  -.2631951   .0627054    -4.20   0.000    -.3877517   -.1386385
------------------------------------------------------------------------------
Note: ATET estimate adjusted for panel effects and time effects.
Note: Treatment occurs at different times.

.
. gen gdp = GDP_2020_const_usd
variable gdp already defined
r(110);

. gen ln_gdp = ln(GDP_2020_const_usd)
variable ln_gdp already defined
r(110);

.
. ssc install xtabond2
checking xtabond2 consistency and verifying not already installed...
all files already exist and are up to date.

.
. *ACM endogenous with covariates restricted lags including time dummies

.
. xtabond2 ln_gdp L.ln_gdp ln_acm urban_pop secd_enroll i.year, gmm(L.ln_gdp ln
> _acm, lag(2 3)) twostep robust small
Favoring space over speed. To switch, type or click on mata: mata set matafavor
>  speed, perm.
1990b.year dropped due to collinearity
1996.year dropped due to collinearity
Warning: Number of instruments may be large relative to number of observations.
Warning: Two-step estimated covariance matrix of moments is singular.
  Using a generalized inverse to calculate optimal weighting matrix for two-ste
> p estimation.
  Difference-in-Sargan/Hansen statistics may be negative.

Dynamic panel-data estimation, two-step system GMM
------------------------------------------------------------------------------
Group variable: country_code                    Number of obs      =      2100
Time variable : year                            Number of groups   =        88
Number of instruments = 182                     Obs per group: min =         1
F(35, 87)     =  2.85e+06                                      avg =     23.86
Prob > F      =     0.000                                      max =        32
------------------------------------------------------------------------------
             |              Corrected
      ln_gdp | Coefficient  std. err.      t    P>|t|     [95% conf. interval]
-------------+----------------------------------------------------------------
      ln_gdp |
         L1. |   1.005344   .0064328   156.28   0.000     .9925578     1.01813
             |
      ln_acm |  -.0506761   .0210379    -2.41   0.018    -.0924911   -.0088611
   urban_pop |   .0012526   .0010689     1.17   0.244    -.0008719     .003377
secd_enroll |  -.0012154   .0006788    -1.79   0.077    -.0025646    .0001337
             |
        year |
       1991  |   -.058486   .0129311    -4.52   0.000     -.084188   -.0327839
       1992  |  -.0303312   .0103109    -2.94   0.004    -.0508252   -.0098372
       1993  |  -.0386567   .0097181    -3.98   0.000    -.0579726   -.0193409
       1994  |  -.0364446   .0136519    -2.67   0.009    -.0635793   -.0093099
       1995  |  -.0119708   .0070813    -1.69   0.095    -.0260458    .0021041
       1997  |  -.0049145   .0063063    -0.78   0.438    -.0174489    .0076199
       1998  |  -.0193849   .0076468    -2.54   0.013    -.0345838    -.004186
       1999  |  -.0241942   .0063613    -3.80   0.000    -.0368381   -.0115504
       2000  |  -.0199595   .0062979    -3.17   0.002    -.0324772   -.0074418
       2001  |  -.0256008   .0069273    -3.70   0.000    -.0393696   -.0118319
       2002  |  -.0282918   .0088555    -3.19   0.002    -.0458932   -.0106905
       2003  |   -.011295    .008833    -1.28   0.204    -.0288515    .0062616
       2004  |   .0080606   .0095475     0.84   0.401    -.0109161    .0270374
       2005  |   .0027828   .0088364     0.31   0.754    -.0147805     .020346
       2006  |   .0072496   .0092898     0.78   0.437    -.0112149    .0257141
       2007  |   .0071736   .0100522     0.71   0.477    -.0128061    .0271534
       2008  |  -.0032633   .0100898    -0.32   0.747     -.023318    .0167913
       2009  |   -.040407   .0129706    -3.12   0.002    -.0661874   -.0146265
       2010  |   .0019592   .0116679     0.17   0.867     -.021232    .0251505
       2011  |  -.0030775   .0110189    -0.28   0.781    -.0249788    .0188238
       2012  |  -.0208687   .0122869    -1.70   0.093    -.0452903    .0035529
       2013  |  -.0152701    .013148    -1.16   0.249    -.0414032    .0108629
       2014  |  -.0090791   .0125739    -0.72   0.472     -.034071    .0159129
       2015  |  -.0129647   .0118258    -1.10   0.276    -.0364698    .0105405
       2016  |  -.0151533   .0123973    -1.22   0.225    -.0397944    .0094877
       2017  |  -.0111099   .0116547    -0.95   0.343     -.034275    .0120551
       2018  |   -.010767   .0127781    -0.84   0.402    -.0361649    .0146309
       2019  |  -.0149763   .0126534    -1.18   0.240    -.0401262    .0101736
       2020  |  -.0867572    .016179    -5.36   0.000    -.1189148   -.0545997
       2021  |   .0087218     .01287     0.68   0.500    -.0168589    .0343024
       2022  |   .0048971   .0138429     0.35   0.724    -.0226172    .0324114
             |
       _cons |   .0520763   .1316557     0.40   0.693    -.2096036    .3137562
------------------------------------------------------------------------------
Instruments for first differences equation
  GMM-type (missing=0, separate instruments for each period unless collapsed)
    L(2/3).(L.ln_gdp ln_acm)
Instruments for levels equation
  Standard
    _cons
  GMM-type (missing=0, separate instruments for each period unless collapsed)
    DL.(L.ln_gdp ln_acm)
------------------------------------------------------------------------------
Arellano-Bond test for AR(1) in first differences: z =  -3.11  Pr > z =  0.002
Arellano-Bond test for AR(2) in first differences: z =  -1.68  Pr > z =  0.092
------------------------------------------------------------------------------
Sargan test of overid. restrictions: chi2(146)  = 440.18  Prob > chi2 =  0.000
  (Not robust, but not weakened by many instruments.)
Hansen test of overid. restrictions: chi2(146)  =  56.90  Prob > chi2 =  1.000
  (Robust, but weakened by many instruments.)

Difference-in-Hansen tests of exogeneity of instrument subsets:
  GMM instruments for levels
    Hansen test excluding group:     chi2(85)   =  48.52  Prob > chi2 =  1.000
    Difference (null H = exogenous): chi2(61)   =   8.39  Prob > chi2 =  1.000
```

**S6. Code for Specification Testing of Models**

use "/Users/Desktop/panel_data_for_ACM_model_W_GDP_20May2025.dta"

encode country, gen(country_code)

duplicates drop country_code year, force

xtset country_code year

generate treated = (period>=1 & pepfar_cop==1)

keep if (pepfar_cop==1 | control==1) & (year<=2022)

drop if country == "India"

*test acm model for FE or RE

xtreg acm urban_pop secd_enroll, fe

estimates store fixed

xtreg acm urban_pop secd_enroll, re

estimates store random

hausman fixed random, sigmamore

*test ln_acm model for FE or RE

gen ln_acm = ln(acm)

xtreg ln_acm urban_pop secd_enroll, fe

estimates store fixed

xtreg ln_acm urban_pop secd_enroll, re

estimates store random

hausman fixed random, sigmamore

*heterogeneous DID treatment effects model for ACM using FE

xthdidregress twfe (acm) (treated), group(country_code) vce(robust)

estat aggregation, cohort

estat aggregation, time

estat aggregation, dynamic graph

*common panel data DID treatment effects model for ACM

xtdidregress (acm) (treated), group(country_code) time(year) vce(robust)

*heterogeneous DID treatment effects model for ln_ACM using FE

xthdidregress twfe (ln_acm) (treated), group(country_code) vce(robust)

estat aggregation, cohort

estat aggregation, time

estat aggregation, dynamic graph

*common panel data DID treatment effects model for ACM

xtdidregress (ln_acm) (treated), group(country_code) time(year) vce(robust)

*ACM model to generated predicted ACM as a function of HIV prevalence

xtreg acm hiv_prevalence, fe

predict acm_hat

gen gdp = GDP_2020_const_usd

gen ln_gdp = ln(GDP_2020_const_usd)

gen ln_acm_hat = ln(acm_hat)

*test ln_gdp models with covariates for fe vs re

xtreg ln_gdp acm urban_pop secd_enroll, fe

estimates store fixed

xtreg ln_gdp acm urban_pop secd_enroll, re

estimates store random

hausman fixed random, sigmamore

xtreg ln_gdp ln_acm urban_pop secd_enroll, fe

estimates store fixed

xtreg ln_gdp ln_acm urban_pop secd_enroll, re

estimates store random

hausman fixed random, sigmamore

*Simplest tests of endogeneity in GDP models without covariates

xtreg ln_gdp acm_hat, fe

estimates store iv

xtreg ln_gdp acm, fe

estimates store fixed

hausman iv fixed, sigmamore

xtreg ln_gdp ln_acm_hat, fe

estimates store iv

xtreg ln_gdp ln_acm, fe

estimates store fixed

hausman iv fixed, sigmamore

*Simplest tests of endogeneity in GDP models with covariates

xtreg ln_gdp acm_hat urban_pop secd_enroll, fe

estimates store iv

xtreg ln_gdp acm urban_pop secd_enroll, fe

estimates store fixed

hausman iv fixed, sigmamore

xtreg ln_gdp ln_acm_hat urban_pop secd_enroll, fe

estimates store iv

xtreg ln_gdp ln_acm urban_pop secd_enroll, fe

estimates store fixed

hausman iv fixed, sigmamore

*test ln_gdp models with covariates for endogeneity using xtivreg

xtivreg ln_gdp (acm = hiv_prevalence)urban_pop secd_enroll, fe

estimates store iv

xtreg ln_gdp acm urban_pop secd_enroll, fe

estimates store fixed

hausman fixed iv, sigmamore

xtivreg ln_gdp (ln_acm = hiv_prevalence) urban_pop secd_enroll, fe

estimates store iv

xtreg ln_gdp ln_acm urban_pop secd_enroll, fe

estimates store fixed

hausman iv fixed, sigmamore

ssc install xtabond2

*xtabond models

xtabond ln_gdp ln_acm urban_pop secd_enroll, vce(robust)

xtabond ln_gdp acm urban_pop secd_enroll, vce(robust)

xtabond ln_gdp ln_acm urban_pop secd_enroll, vce(robust) twostep

xtabond ln_gdp acm urban_pop secd_enroll, vce(robust) twostep

xtabond ln_gdp ln_acm urban_pop secd_enroll, lags(2)

xtabond ln_gdp ln_acm urban_pop secd_enroll i.year, lags(2)

xtabond ln_gdp acm urban_pop secd_enroll, lags(2)

*GDP ACM Models

*ACM exogenous without covariates restricted lags no time dummies

xtabond2 gdp L.gdp acm, gmm(L.gdp, lag(2 3)) iv(acm) twostep robust small

*ACM endogenous without covariates restricted lags no time dummies

xtabond2 gdp L.gdp acm, gmm(L.gdp acm, lag(2 3)) twostep robust small

*ACM exogenous without covariates restricted lags including time dummies

xtabond2 gdp L.gdp acm i.year, gmm(L.gdp, lag(2 3)) iv(acm) twostep robust small

*ACM endogenous without covariates restricted lags including time dummies

xtabond2 gdp L.gdp acm i.year, gmm(L.gdp acm, lag(2 3)) twostep robust small

*ACM exogenous with covariates restricted lags including time dummies

xtabond2 gdp L.gdp acm urban_pop secd_enroll i.year, gmm(L.gdp, lag(2 3)) iv(acm) twostep robust small

*ACM endogenous with covariates restricted lags including time dummies

xtabond2 gdp L.gdp acm urban_pop secd_enroll i.year, gmm(L.gdp acm, lag(2 3)) twostep robust small

*ACM exogenous without covariates unrestricted lags no time dummies

xtabond2 gdp L.gdp acm, gmm(L.gdp) iv(acm) twostep robust small

*ACM endogenous without covariates unrestricted lags no time dummies

xtabond2 gdp L.gdp acm, gmm(L.gdp acm) twostep robust small

*ACM exogenous without covariates unrestricted lags including time dummies

xtabond2 gdp L.gdp acm i.year, gmm(L.gdp) iv(acm) twostep robust small

*ACM endogenous without covariates unrestricted lags including time dummies

xtabond2 gdp L.gdp acm i.year, gmm(L.gdp acm) twostep robust small

*ACM exogenous with covariates unrestricted lags including time dummies

xtabond2 gdp L.gdp acm urban_pop secd_enroll i.year, gmm(L.gdp) iv(acm) twostep robust small

*ACM endogenous with covariates unrestricted lags including time dummies

xtabond2 gdp L.gdp acm urban_pop secd_enroll i.year, gmm(L.gdp acm) twostep robust small

*ln_GDP ACM Models

*ACM exogenous without covariates restricted lags no time dummies

xtabond2 ln_gdp L.ln_gdp acm, gmm(L.ln_gdp, lag(2 3)) iv(acm) twostep robust small

*ACM endogenous without covariates restricted lags no time dummies

xtabond2 ln_gdp L.ln_gdp acm, gmm(L.ln_gdp acm, lag(2 3)) twostep robust small

*ACM exogenous without covariates restricted lags including time dummies

xtabond2 ln_gdp L.ln_gdp acm i.year, gmm(L.ln_gdp, lag(2 3)) iv(acm) twostep robust small

*ACM endogenous without covariates restricted lags including time dummies

xtabond2 ln_gdp L.gdp acm i.year, gmm(L.ln_gdp acm, lag(2 3)) twostep robust small

*ACM exogenous with covariates restricted lags including time dummies

xtabond2 ln_gdp L.ln_gdp acm urban_pop secd_enroll i.year, gmm(L.ln_gdp, lag(2 3)) iv(acm) twostep robust small

*ACM endogenous with covariates restricted lags including time dummies

xtabond2 ln_gdp L.ln_gdp acm urban_pop secd_enroll i.year, gmm(L.ln_gdp acm, lag(2 3)) twostep robust small

*ACM exogenous without covariates unrestricted lags no time dummies

xtabond2 ln_gdp L.ln_gdp acm, gmm(L.ln_gdp) iv(acm) twostep robust small

*ACM endogenous without covariates unrestricted lags no time dummies

xtabond2 ln_gdp L.ln_gdp acm, gmm(L.ln_gdp acm) twostep robust small

*ACM exogenous without covariates unrestricted lags including time dummies

xtabond2 ln_gdp L.ln_gdp acm i.year, gmm(L.ln_gdp) iv(acm) twostep robust small

*ACM endogenous without covariates unrestricted lags including time dummies

xtabond2 ln_gdp L.ln_gdp acm i.year, gmm(L.ln_gdp acm) twostep robust small

*ACM exogenous with covariates unrestricted lags including time dummies

xtabond2 ln_gdp L.ln_gdp acm urban_pop secd_enroll i.year, gmm(L.ln_gdp) iv(acm) twostep robust small

*ACM endogenous with covariates unrestricted lags including time dummies

xtabond2 ln_gdp L.ln_gdp acm urban_pop secd_enroll i.year, gmm(L.ln_gdp acm) twostep robust small

*ln_GDP Ln_ACM Models

*ACM exogenous without covariates restricted lags no time dummies

xtabond2 ln_gdp L.ln_gdp ln_acm, gmm(L.ln_gdp, lag(2 3)) iv(ln_acm) twostep robust small

*ACM endogenous without covariates restricted lags no time dummies

xtabond2 ln_gdp L.ln_gdp ln_acm, gmm(L.ln_gdp ln_acm, lag(2 3)) twostep robust small

*ACM exogenous without covariates restricted lags including time dummies

xtabond2 ln_gdp L.ln_gdp ln_acm i.year, gmm(L.ln_gdp, lag(2 3)) iv(ln_acm) twostep robust small

*ACM endogenous without covariates restricted lags including time dummies

xtabond2 ln_gdp L.gdp ln_acm i.year, gmm(L.ln_gdp ln_acm, lag(2 3)) twostep robust small

*ACM exogenous with covariates restricted lags including time dummies

xtabond2 ln_gdp L.ln_gdp ln_acm urban_pop secd_enroll i.year, gmm(L.ln_gdp, lag(2 3)) iv(ln_acm) twostep robust small

*ACM endogenous with covariates restricted lags including time dummies

xtabond2 ln_gdp L.ln_gdp ln_acm urban_pop secd_enroll i.year, gmm(L.ln_gdp ln_acm, lag(2 3)) twostep robust small

*ACM exogenous without covariates unrestricted lags no time dummies

xtabond2 ln_gdp L.ln_gdp ln_acm, gmm(L.ln_gdp) iv(ln_acm) twostep robust small

*ACM endogenous without covariates unrestricted lags no time dummies

xtabond2 ln_gdp L.ln_gdp ln_acm, gmm(L.ln_gdp ln_acm) twostep robust small

*ACM exogenous without covariates unrestricted lags including time dummies

xtabond2 ln_gdp L.ln_gdp ln_acm i.year, gmm(L.ln_gdp) iv(ln_acm) twostep robust small

*ACM endogenous without covariates unrestricted lags including time dummies

xtabond2 ln_gdp L.ln_gdp ln_acm i.year, gmm(L.ln_gdp ln_acm) twostep robust small

*ACM exogenous with covariates unrestricted lags including time dummies

xtabond2 ln_gdp L.ln_gdp ln_acm urban_pop secd_enroll i.year, gmm(L.ln_gdp) iv(ln_acm) twostep robust small

*ACM endogenous with covariates unrestricted lags including time dummies

xtabond2 ln_gdp L.ln_gdp ln_acm urban_pop secd_enroll i.year, gmm(L.ln_gdp ln_acm) twostep robust small

**S7. Parallel Trends Test for ACM DID Model**

We tested the parallel trend of ACM for the COP PEPFAR countries and the non-PEPFAR countries. To do this, we regressed ACM on three variables – a dummy variable indicating countries’ PEPFAR status, a year categorical variable (with year 1990 as the reference year), and an interaction between the two. The coefficient of the interaction term indicates the difference in ACM trend between the PEPFAR and non-PEPFAR countries. A statistically insignificant coefficient of the interaction term, where its 95% confidence interval cross 0, means that there is no statistically significant difference in ACM trend between the two groups, and therefore, the parallel assumption holds between all PEPFAR and the control countries. Our tests indicate that the parallel assumption holds for all country cohorts before 2004.

**S8. Listing of COP and Comparison Group Countries**

**PEPFAR Country Operational Plan (COP) Countries**

Angola, Botswana, Burundi, Cambodia, Cameroon, Dem. Rep. Congo, Cote d'Ivoire, Dominican Republic, Eswatini, Ethiopia, Ghana, Guyana, Haiti, India, Indonesia, Kenya, Lesotho, Malawi, Mozambique, Myanmar, Namibia, Nigeria, Papua New Guinea, Rwanda, South Africa, Tanzania, Uganda, Ukraine, Vietnam, Zambia, Zimbabwe

**Control group (non=COP LMICs)**

Algeria, American Samoa, Antigua and Barbuda, Argentina, Azerbaijan, Belarus, Bhutan, Bosnia and Herzegovina, Bulgaria, Cabo Verde, Chile, Colombia, Costa Rica, Croatia, Cuba, Czech Republic, Dominica, Ecuador, Arab Ep. Egypt, Equatorial Guinea, Fiji, Grenada, Hungary, Islamic Rep. Iran, Iraq, Kiribati, Dem. People’s Rep. Korea, Kosovo, Latvia, Lebanon, Libya, Lithuania, Malaysia, Maldives, Marshall Islands, Mauritius, Mayotte, Fed. Sts. Micronesia, Mongolia, Montenegro, Morocco, Nauru, Northern Mariana Islands, Oman, Palau, Panama, Paraguay, Poland, Serbia and Montenegro, Serbia, Slovak Republic, Solomon Islands, Somalia, Sri Lanka, St. Kitts and Nevis, St. Lucia, St. Vincent and the Grenadines, Syrian Arab Republic, Tonga, Tunisia, Turkey, Tuvalu, Uruguay, Vanuatu, Venezuela, West Bank and Gaza, Rep. Yemen

**S9. Country-level Dynamic Panel Estimation – Benefits from 2004 to 2022 (reported in 2022 dollars)**

| **Country** | **2004 GDP** | **Estimated Economic Benefits from PEPEFAR from 2004 to 2022** |
| --- | --- | --- |
| Angola | $ 48,402,700,000 | $ 27,097,520,510 |
| Botswana | $ 11,085,100,000 | $ 4,302,360,310 |
| Burundi | $ 2,465,713,203 | $ 1,211,853,168 |
| Cambodia | $ 9,323,550,830 | $ 5,068,363,339 |
| Cameroon | $ 24,132,400,000 | $ 9,352,810,020 |
| Cote d'Ivoire | $ 32,500,500,000 | $ 14,457,763,530 |
| DRC | $ 21,445,300,000 | $ 50,003,189,440 |
| Dominican Republic | $ 44,134,200,000 | $ 19,748,190,755 |
| Eswatini | $ 3,223,362,729 | $ 1,760,685,851 |
| Ethiopia | $ 24,217,500,000 | $ 18,387,676,176 |
| Ghana | $ 27,670,800,000 | $ 14,250,853,840 |
| Guyana | $ 3,429,172,754 | $ 1,379,801,315 |
| Haiti | $ 13,099,500,000 | $ 4,307,386,780 |
| Indonesia | $ 537,876,000,000 | $ 304,116,028,500 |
| Kenya | $ 47,241,200,000 | $ 24,463,497,360 |
| Lesotho | $ 1,841,522,156 | $ 1,382,216,445 |
| Malawi | $ 5,827,510,571 | $ 2,819,085,453 |
| Mozambique | $ 8,546,393,549 | $ 7,098,131,562 |
| Myanmar | $ 24,913,900,000 | $ 16,040,226,960 |
| Namibia | $ 7,949,134,446 | $ 4,274,518,248 |
| Nigeria | $ 294,055,000,000 | $ 125,431,715,580 |
| Papua New Guinea | $ 13,170,400,000 | $ 7,201,887,240 |
| Rwanda | $ 4,218,113,907 | $ 36,148,694,770 |
| South Africa | $ 290,325,000,000 | $ 92,829,892,110 |
| Tanzania | $ 27,511,100,000 | $ 13,579,290,050 |
| Uganda | $ 18,158,000,000 | $ 8,949,582,484 |
| Ukraine | $ 111,950,000,000 | $ 29,147,690,380 |
| Vietnam | $ 138,673,000,000 | $ 67,859,589,800 |
| Zambia | $ 11,598,900,000 | $ 5,647,775,160 |
| Zimbabwe | $ 16,377,800,000 | $ 5,699,121,310 |

*Note: Country level estimates were derived by applying the average annual PEPFAR impact on GDP (percent ACM reduction multiplied by the GDP coefficient), then multiplying the annual impact by country specific GDP; estimated economic benefit is the total benefit for each respective country from 2004 to 2022

**S10. Country Year Results through 2022 – all COP Countries**

```
       country      year   lives saved  population   acm   gdp_pc_currt
  1.   Angola       2004   59951.27     18600423   15.151   4224.139738
  2.   Angola       2005   62177.59     19291161   14.497   4836.226882
  3.   Angola       2006   64511.51     20015279   13.943   5364.965087
  4.   Angola       2007   66971.65     20778561   13.133    6057.77173
  5.   Angola       2008   69550.45     21578655   12.478   6616.768779
  6.   Angola       2009   72245.34     22414773   11.788   6471.347922
  7.   Angola       2010   75081.85     23294825   11.177   6587.408285
  8.   Angola       2011   78058.48     24218352    10.63     6700.1141
  9.   Angola       2012   81149.59     25177394   10.031   7351.760767
 10.   Angola       2013   84334.75     26165620    9.607   7566.423051
 11.   Angola       2014   87542.23     27160769    9.181   7999.826851
 12.   Angola       2015   90755.77     28157798    8.826   7127.339573
 13.   Angola       2016   94060.34     29183070    8.555   6850.384625
 14.   Angola       2017    97450.3     30234839    8.242   6998.796187
 15.   Angola       2018   100874.3     31297155     7.99   7353.349988
 16.   Angola       2019   104350.3     32375632    7.811   7533.511854
 17.   Angola       2020   107816.8     33451132    7.821   6455.119982
 18.   Angola       2021   111301.9     34532429    8.009   7414.278958
 19.   Angola       2022   114855.7     35635029    7.863   7928.641498
 20.   Botswana     2004   5824.636      1807146   13.073   10299.53078
 21.   Botswana     2005   5930.131      1839877   12.568   10908.62423
 22.   Botswana     2006   6041.777      1874516   11.883    11958.7509
 23.   Botswana     2007   6160.017      1911201   10.935   12741.39038
 24.   Botswana     2008   6284.974      1949970   10.253   13139.72502
 25.   Botswana     2009   6416.164      1990673    9.719   11119.91449
 26.   Botswana     2010   6552.943      2033110     8.93   12141.48683
 27.   Botswana     2011   6683.238      2073535    8.738    12976.5292
 28.   Botswana     2012   6796.308      2108616    8.671   12091.68763
 29.   Botswana     2013    6899.66      2140682    8.157   12880.30526
 30.   Botswana     2014   7000.743      2172044    7.927   14417.01321
 31.   Botswana     2015   7101.398      2203273    7.435   13518.48486
 32.   Botswana     2016   7202.936      2234776    6.783   15416.58452
 33.   Botswana     2017   7305.982      2266747    6.308   14817.57128
 34.   Botswana     2018   7410.391      2299141    6.922   14805.29163
 35.   Botswana     2019   7516.567      2332083    6.974    14889.8073
 36.   Botswana     2020   7625.544      2365894     6.95   14208.27016
 37.   Botswana     2021   7740.116      2401441    9.461   16663.86854
 38.   Botswana     2022   7864.048      2439892    6.897   18516.19134
 39.   Burundi      2004   23563.07      7310656   13.369   566.1589076
 40.   Burundi      2005   24454.72      7587299   12.841   567.7661336
 41.   Burundi      2006   25351.55      7865547   12.366   595.2640856
 42.   Burundi      2007   26306.64      8161874   11.871   609.6934915
 43.   Burundi      2008   27414.63      8505637   11.316   625.4000522
 44.   Burundi      2009   28839.27      8947646    10.92   620.9027826
 45.   Burundi      2010   30221.34      9376444   10.538   630.4483917
 46.   Burundi      2011   31322.14      9717978   10.094   646.1031275
 47.   Burundi      2012   32460.06     10071028    9.625   656.8448261
 48.   Burundi      2013   33647.17     10439340    9.178   706.9897821
 49.   Burundi      2014   34808.93     10799786    8.798   745.4026654
 50.   Burundi      2015    35607.6     11047580    8.553   821.2462547
 51.   Burundi      2016   36226.02     11239451     8.18   795.7859958
 52.   Burundi      2017   37087.59     11506762    7.772   785.2333947
 53.   Burundi      2018   38224.34     11859446    7.648   777.4469722
 54.   Burundi      2019   39500.33     12255336    7.352   804.1653441
 55.   Burundi      2020   40666.13     12617036    7.562    812.055173
 56.   Burundi      2021   41789.21     12965482    7.474   864.2807327
 57.   Burundi      2022    42935.4     13321098    7.311    917.506035
 58.   Cambodia     2004   42689.27     13244731    7.273   1505.462904
 59.   Cambodia     2005   43316.07     13439202    7.042   1727.836497
 60.   Cambodia     2006   43960.13     13639028    6.809   1939.122101
 61.   Cambodia     2007   44613.59     13841770     6.63    2157.12361
 62.   Cambodia     2008   45295.95     14053480    6.418   2307.270331
 63.   Cambodia     2009   46015.77     14276810    6.127   2288.740553
 64.   Cambodia     2010   46737.48     14500726    6.121   2419.182169
 65.   Cambodia     2011   47452.55     14722584    5.947   2605.489544
 66.   Cambodia     2012    48169.7     14945086    5.863   2855.491165
 67.   Cambodia     2013   48895.29     15170208    5.817   3025.421459
 68.   Cambodia     2014   49625.54     15396772    5.756    3158.53082
 69.   Cambodia     2015    50355.5     15623250     5.81   3354.610833
 70.   Cambodia     2016   51095.38     15852803    5.779   3631.128525
 71.   Cambodia     2017   51806.29     16073372    5.775   3873.636032
 72.   Cambodia     2018   52454.63     16274522    5.874   4228.760962
 73.   Cambodia     2019   53121.11     16481304    5.947   4620.963392
 74.   Cambodia     2020   53908.09     16725474    6.228    4504.24716
 75.   Cambodia     2021    54710.1     16974305    6.838    4668.23778
 76.   Cambodia     2022    55443.1     17201724    6.785   5202.677725
 77.   Cameroon     2004   53537.48     16610487   12.565   2502.676474
 78.   Cameroon     2005   55033.35     17074594   12.408   2567.519485
 79.   Cameroon     2006   56566.95     17550407   12.104   2673.826075
 80.   Cameroon     2007   58176.86     18049896   11.886   2786.542161
 81.   Cameroon     2008   59866.28     18574056   11.583   2839.404759
 82.   Cameroon     2009    61606.5     19113974     11.3   2848.357705
 83.   Cameroon     2010    63392.4     19668066   10.992   2883.163197
 84.   Cameroon     2011      65211     20232304   10.641   2957.163456
 85.   Cameroon     2012   67071.46     20809529   10.235    3027.17972
 86.   Cameroon     2013   68982.27     21402376    9.833   3204.361753
 87.   Cameroon     2014   71101.53     22059893    9.548   3385.476353
 88.   Cameroon     2015   73369.05     22763414    9.179   3459.780244
 89.   Cameroon     2016   75595.41     23454161    8.886   3587.616119
 90.   Cameroon     2017   77769.21     24128601    8.594    3726.56739
 91.   Cameroon     2018   79953.77     24806383    8.379   3967.521705
 92.   Cameroon     2019   82209.02     25506095     8.16   4195.670175
 93.   Cameroon     2020   84479.59     26210558    8.419   4319.218871
 94.   Cameroon     2021   86752.53     26915758    8.582    4741.76177
 95.   Cameroon     2022   89063.55     27632771    8.225   5122.644393
 96.   Cote d'I..   2004    63156.9     19595000   13.465   2952.392093
 97.   Cote d'I..   2005   64682.91     20068458   13.117   3006.344803
 98.   Cote d'I..   2006   66224.66     20546802   12.753    3116.67755
 99.   Cote d'I..   2007   67783.87     21030558   12.364   3167.190469
100.   Cote d'I..   2008   69343.47     21514439   11.952   3311.302978
101.   Cote d'I..   2009   70901.84     21997940   11.529   3379.445487
102.   Cote d'I..   2010   72481.58     22488065   11.187   3578.218921
103.   Cote d'I..   2011   74073.59     22982000   10.927   3384.930979
104.   Cote d'I..   2012   75637.05     23467078   10.517   3508.457834
105.   Cote d'I..   2013    77160.6     23939775   10.144   3855.865463
106.   Cote d'I..   2014   79054.56     24527395    9.847   4345.616675
107.   Cote d'I..   2015   81371.81     25246342    9.543   4711.492844
108.   Cote d'I..   2016   83766.79     25989405    9.374   4862.918019
109.   Cote d'I..   2017   86164.68     26733372     9.17   5046.047689
110.   Cote d'I..   2018   88520.13     27464173    8.961   5327.824985
111.   Cote d'I..   2019   90869.25     28193009    8.728   5947.924089
112.   Cote d'I..   2020   93197.76     28915449    8.817   5978.822741
113.   Cote d'I..   2021   95532.22     29639736    9.001    6520.73116
114.   Cote d'I..   2022   97966.52     30395002    8.843   7234.226693
115.   Democrat..   2004   183710.2     56997741   13.465   474.0152567
116.   Democrat..   2005   189440.8     58775724   13.136   502.9551638
117.   Democrat..   2006   195371.9     60615908   12.777   528.9236978
118.   Democrat..   2007   201372.9     62477752   12.529   558.9917424
119.   Democrat..   2008   207538.4     64390664   12.275   586.1752581
120.   Democrat..   2009   214053.5     66412044    11.98   587.5585393
121.   Democrat..   2010   220986.4     68563038   11.653   616.6206195
122.   Democrat..   2011   228355.3     70849311   11.301   650.4366032
123.   Democrat..   2012   236107.9     73254618   11.166   652.2973377
124.   Democrat..   2013   244277.8     75789395   10.857    735.030028
125.   Democrat..   2014   252702.5     78403242   10.551   829.6507769
126.   Democrat..   2015   261186.7     81035531   10.396   885.1244428
127.   Democrat..   2016     270601     83956415   10.077   942.3002777
128.   Democrat..   2017   280679.6     87083398    9.865   1036.588358
129.   Democrat..   2018   290233.7     90047644    9.573   1105.628847
130.   Democrat..   2019   299580.1     92947442     9.38   1206.798113
131.   Democrat..   2020   309386.6     95989998    9.537   1174.385868
132.   Democrat..   2021   319568.2     99148932      9.7   1358.204717
133.   Democrat..   2022     330037    102396968    9.352   1533.743878
134.   Dominica..   2004   29147.02      9043127    6.089   7452.720836
135.   Dominica..   2005   29539.08      9164768    5.933   8299.451614
136.   Dominica..   2006   29923.92      9284168    5.873   9220.173495
137.   Dominica..   2007   30304.37      9402206    5.859   10044.55488
138.   Dominica..   2008   30693.54      9522948    5.831   10432.70056
139.   Dominica..   2009   31096.79      9648061    5.831   10458.95548
140.   Dominica..   2010   31508.36      9775755    5.821   11319.09197
141.   Dominica..   2011   31920.86      9903737     5.84   11760.64189
142.   Dominica..   2012   32330.67     10030882    5.802   11846.43857
143.   Dominica..   2013   32737.32     10157051    5.803   12484.10743
144.   Dominica..   2014   33140.42     10282115    5.827   13541.93457
145.   Dominica..   2015   33539.17     10405832    5.883   14886.77901
146.   Dominica..   2016   33931.62     10527592    5.964   16351.83448
147.   Dominica..   2017   34317.27     10647244    6.032   17075.24595
148.   Dominica..   2018   34698.52     10765531     6.06   18141.33826
149.   Dominica..   2019   35073.54     10881882    6.006   19743.40045
150.   Dominica..   2020   35453.16     10999664     6.46   19085.35362
151.   Dominica..   2021   35834.16     11117873    6.678   21923.30194
152.   Dominica..   2022   36191.76     11228821    6.068   24363.89337
153.   Eswatini     2004   3458.909      1073158   18.372   5494.454546
154.   Eswatini     2005    3477.81      1079022   18.845   5972.358545
155.   Eswatini     2006    3495.26      1084436   18.174   6490.031863
156.   Eswatini     2007   3515.466      1090705   17.861   6921.234365
157.   Eswatini     2008   3539.275      1098092   17.525   7074.295716
158.   Eswatini     2009   3562.033      1105153   17.283   7196.207957
159.   Eswatini     2010   3582.974      1111650   15.911   7525.387057
160.   Eswatini     2011   3602.902      1117833   14.926   7814.518442
161.   Eswatini     2012   3622.337      1123863   13.946   8076.337181
162.   Eswatini     2013   3642.085      1129990   13.019   8567.039897
163.   Eswatini     2014    3662.42      1136299   12.193   8715.031268
164.   Eswatini     2015   3683.412      1142812   11.202   8792.214728
165.   Eswatini     2016   3704.991      1149507    10.45   8750.877727
166.   Eswatini     2017   3732.993      1158195    9.936   8519.584386
167.   Eswatini     2018    3767.59      1168929    9.324   8640.083598
168.   Eswatini     2019   3802.867      1179874    8.923   9083.437861
169.   Eswatini     2020     3844.3      1192729    9.603    9077.23198
170.   Eswatini     2021   3888.986      1206593   11.356   10202.50961
171.   Eswatini     2022   3928.707      1218917   11.883   10887.00387
172.   Ethiopia     2004   245205.4     76077206   12.745   563.5755313
173.   Ethiopia     2005   252587.2     78367470   12.226   631.7459772
174.   Ethiopia     2006   260116.5     80703490   11.539   701.6694792
175.   Ethiopia     2007   267851.1     83103218   10.807   780.6604652
176.   Ethiopia     2008   275716.9     85543668   10.143    856.873946
177.   Ethiopia     2009   283658.6     88007636    9.485   912.1172452
178.   Ethiopia     2010   291815.9     90538514    9.001   1010.166552
179.   Ethiopia     2011   300232.9     93149970     8.52   1114.045972
180.   Ethiopia     2012   308770.3     95798767    8.119   1195.794561
181.   Ethiopia     2013   317279.2     98438751    7.734   1270.347236
182.   Ethiopia     2014     325907    101115610    7.502   1505.005338
183.   Ethiopia     2015   334775.5    103867134    7.208   1654.847633
184.   Ethiopia     2016   343964.7    106718162     6.93   1882.687154
185.   Ethiopia     2017   353467.5    109666481    6.767     2031.9573
186.   Ethiopia     2018   363129.3    112664152    6.569   2124.254622
187.   Ethiopia     2019   373034.7    115737383    6.439   2274.214139
188.   Ethiopia     2020   383285.1    118917671    6.616   2442.252484
189.   Ethiopia     2021   393666.5    122138588    6.776   2628.343464
190.   Ethiopia     2022   404127.7    125384287    6.497   2888.736492
191.   Ghana        2004   70483.99     21868298    9.476   2724.591856
192.   Ghana        2005   72357.08     22449438    9.247   2897.701465
193.   Ghana        2006   74262.79     23040702     9.14   3095.449715
194.   Ghana        2007   76201.73     23642279    9.026   3232.192189
195.   Ghana        2008   78160.31     24249946    8.905   3504.585391
196.   Ghana        2009   80135.17     24862664    8.706   3604.467427
197.   Ghana        2010   82108.79     25474995    8.626   3840.435212
198.   Ghana        2011   84107.41     26095088    8.417   4362.592882
199.   Ghana        2012   86167.57     26734268    8.238   4910.784396
200.   Ghana        2013    88268.8     27386193    8.092   5188.160056
201.   Ghana        2014   90381.22     28041592    7.817   5486.848997
202.   Ghana        2015   92490.67     28696068    7.773   5159.926452
203.   Ghana        2016   94620.09     29356742     7.46   4977.581864
204.   Ghana        2017   96720.31     30008354     7.39   5134.361772
205.   Ghana        2018    98748.4     30637585    7.373   5494.700504
206.   Ghana        2019   100751.1     31258945    7.143   5952.403657
207.   Ghana        2020     102778     31887809    7.448    5992.31759
208.   Ghana        2021   104811.3     32518665    7.627     6544.2382
209.   Ghana        2022   106843.5     33149152    7.585   7132.783653
210.   Guyana       2004   2450.932       760424    7.402   6690.320613
211.   Guyana       2005   2448.628       759709    7.414   6771.842021
212.   Guyana       2006   2444.302       758367    7.297    7351.74251
213.   Guyana       2007   2438.353       756521     7.16   8113.674855
214.   Guyana       2008   2430.711       754150    7.288   8441.615559
215.   Guyana       2009   2421.389       751258    7.321   8834.309495
216.   Guyana       2010   2410.669       747932    7.399   9353.126083
217.   Guyana       2011   2398.737       744230    7.455   10092.08249
218.   Guyana       2012   2397.886       743966    7.513   10659.99316
219.   Guyana       2013   2409.019       747420    7.538   11238.86276
220.   Guyana       2014   2420.928       751115    7.459   11191.23459
221.   Guyana       2015    2433.55       755031    7.431   11465.13618
222.   Guyana       2016   2446.623       759087    7.475   11584.05925
223.   Guyana       2017   2460.047       763252    7.472    12327.0682
224.   Guyana       2018     2531.8       785514    7.499   12538.21374
225.   Guyana       2019   2574.471       798753    7.444   13388.70608
226.   Guyana       2020   2569.472       797202    8.026   17036.28992
227.   Guyana       2021    2593.21       804567    9.987    23176.1332
228.   Guyana       2022   2606.615       808726    9.762   40337.59901
229.   Haiti        2004   28883.89      8961489   10.184   2308.923633
230.   Haiti        2005   29368.68      9111900    9.239   2413.960842
231.   Haiti        2006   29866.29      9266288    9.084   2490.223854
232.   Haiti        2007   30364.39      9420826    8.937   2634.138229
233.   Haiti        2008    30862.1      9575247    8.853    2711.80526
234.   Haiti        2009   31362.95      9730638    8.695   2843.112786
235.   Haiti        2010   31724.71      9842880   17.738   2684.072674
236.   Haiti        2011   32083.87      9954312    8.762   2846.881182
237.   Haiti        2012   32580.96     10108539    8.486   2819.336533
238.   Haiti        2013   33073.03     10261206    8.376   3030.979635
239.   Haiti        2014   33561.44     10412740    8.243   3064.069363
240.   Haiti        2015   34048.18     10563757    8.181   3014.229685
241.   Haiti        2016   34531.95     10713849    8.161   3240.598619
242.   Haiti        2017   35014.43     10863543    8.014   3325.313579
243.   Haiti        2018   35494.28     11012421    7.998   3182.441427
244.   Haiti        2019   35971.35     11160438    7.953   3220.942957
245.   Haiti        2020    36443.1     11306801    8.176    3085.90305
246.   Haiti        2021   36896.81     11447569    8.676   3114.963542
247.   Haiti        2022   37339.75     11584996     8.48   3239.319005
248.   India        2004    3661429   1135991513     8.04   2681.216657
249.   India        2005    3721652   1154676322    7.876   2936.898878
250.   India        2006    3780321   1172878890    7.743   3222.014495
251.   India        2007    3837683   1190676021    7.642   3510.963992
252.   India        2008    3893298   1207930964    7.558   3636.969354
253.   India        2009    3950005   1225524753    7.479   3892.567908
254.   India        2010    4007882   1243481564    7.385   4216.183993
255.   India        2011    4065071   1261224954    7.267   4467.466655
256.   India        2012    4121313   1278674502    7.118   4835.477665
257.   India        2013    4176605   1295829511     6.96   5032.593975
258.   India        2014    4229618   1312277191     6.79   5211.570321
259.   India        2015    4280373   1328024498     6.67   5446.188766
260.   India        2016    4331685   1343944296    6.603   5823.481542
261.   India        2017    4382330   1359657400    6.593   6169.499918
262.   India        2018    4430682   1374659064    6.644   6742.707913
263.   India        2019    4477002   1389030312     6.71   7181.522654
264.   India        2020    4520796   1402617695     7.35   6997.358393
265.   India        2021    4558139   1414203896    9.448   8087.993803
266.   India        2022    4594300   1425423212    9.074   9199.567739
267.   Indonesia    2004   734633.4    227926649    7.995   5750.204743
268.   Indonesia    2005   744125.4    230871650    7.336    6189.56886
269.   Indonesia    2006   754052.6    233951652    7.323    6644.53394
270.   Indonesia    2007   764078.7    237062338    7.314   7162.984355
271.   Indonesia    2008   774056.1    240157903     7.41   7639.919098
272.   Indonesia    2009   783925.6    243220028    7.386   7941.243696
273.   Indonesia    2010   793869.9    246305322    7.406   8431.821765
274.   Indonesia    2011   804070.1    249470032    7.456    9022.72138
275.   Indonesia    2012   814475.9    252698525    7.494   9624.588232
276.   Indonesia    2013   824641.4    255852467    7.457   9966.382935
277.   Indonesia    2014   834391.1    258877399     7.43   10168.67606
278.   Indonesia    2015   843808.6    261799249     7.46   10132.31608
279.   Indonesia    2016   852924.1    264627418    7.529   10371.44233
280.   Indonesia    2017   861688.5    267346658    7.579    10802.7126
281.   Indonesia    2018   870085.3    269951846    7.496   11494.94485
282.   Indonesia    2019   878264.1    272489381    7.539   12115.70207
283.   Indonesia    2020   885759.4    274814866    8.964   11856.94333
284.   Indonesia    2021   892022.5    276758053   10.065   12897.10314
285.   Indonesia    2022   898702.3    278830529     9.59   14445.64316
286.   Kenya        2004   111885.4     34713455     9.61   2061.660295
287.   Kenya        2005   115376.1     35796484    9.147   2185.854575
288.   Kenya        2006   118945.8     36904008    8.748   2328.789645
289.   Kenya        2007   122595.5     38036349    8.457     2481.0581
290.   Kenya        2008   126366.5     39206351    8.087   2460.342681
291.   Kenya        2009   130242.4     40408891    7.718   2482.775173
292.   Kenya        2010   134076.9     41598566     7.57   2640.012483
293.   Kenya        2011   137815.3     42758461    7.428    2758.24151
294.   Kenya        2012     141457     43888303    7.453   2857.851916
295.   Kenya        2013   144997.5     44986791    7.399   3119.877367
296.   Kenya        2014     148429     46051440    7.304   3375.584568
297.   Kenya        2015   151771.6     47088526    7.361   3696.936075
298.   Kenya        2016   155159.7     48139687     7.34   3936.689762
299.   Kenya        2017     158570     49197766    7.294   4198.553356
300.   Kenya        2018   161823.2     50207107    7.305   4433.975323
301.   Kenya        2019   165032.5     51202828     7.29    4710.50374
302.   Kenya        2020   168302.4     52217334    7.456    4813.92014
303.   Kenya        2021   171531.4     53219166    8.056   5360.361903
304.   Kenya        2022   174861.8     54252460     7.83   5902.091974
305.   Lesotho      2004   6353.171      1971129   20.162   1549.542644
306.   Lesotho      2005   6294.985      1953076   20.605   1660.177062
307.   Lesotho      2006   6269.155      1945062   20.884   1784.348091
308.   Lesotho      2007   6295.604      1953268   20.662   1903.006689
309.   Lesotho      2008   6338.384      1966541   20.417     2034.9595
310.   Lesotho      2009    6384.61      1980883   19.933   2007.595643
311.   Lesotho      2010   6434.143      1996251   18.644   2124.714206
312.   Lesotho      2011   6491.378      2014009   17.828   2252.036207
313.   Lesotho      2012   6556.953      2034354   17.019   2268.784986
314.   Lesotho      2013   6628.345      2056504   16.222   2516.924666
315.   Lesotho      2014   6704.362      2080089   15.519   2751.203047
316.   Lesotho      2015   6783.402      2104612   14.825   3014.721737
317.   Lesotho      2016   6866.591      2130422   14.126   2923.723188
318.   Lesotho      2017   6952.609      2157110   13.651   2602.522208
319.   Lesotho      2018   7037.999      2183603   13.259   2615.025922
320.   Lesotho      2019   7121.162      2209405   13.014   2549.535277
321.   Lesotho      2020   7206.001      2235727   12.858   2537.792082
322.   Lesotho      2021   7289.206      2261542   14.186   2516.977753
323.   Lesotho      2022   7368.391      2286110   14.069   2699.990941
324.   Malawi       2004   40291.29     12500737   15.541   1040.241089
325.   Malawi       2005   41384.87     12840031   14.633   1078.020519
326.   Malawi       2006   42540.91     13198701   13.951   1131.329257
327.   Malawi       2007   43758.09     13576343   13.496   1237.938778
328.   Malawi       2008   45047.57     13976418    12.96   1319.664038
329.   Malawi       2009   46399.25     14395788   12.183   1397.190323
330.   Malawi       2010   47787.37     14826463   11.412    1468.30528
331.   Malawi       2011   49200.89     15265022   10.608    1528.11656
332.   Malawi       2012   50633.74     15709577    9.783   1476.100989
333.   Malawi       2013   52091.36     16161816    9.081   1571.436944
334.   Malawi       2014   53573.09     16621538    8.376    1518.66973
335.   Malawi       2015   55068.78     17085587    8.005   1415.231394
336.   Malawi       2016   56590.58     17557739    7.541   1422.280238
337.   Malawi       2017   58144.04     18039715    7.149   1392.470808
338.   Malawi       2018    59718.1     18528081    6.955    1375.89008
339.   Malawi       2019   61322.15     19025752    6.611   1465.550469
340.   Malawi       2020   62959.93     19533888    6.706   1560.961347
341.   Malawi       2021   64614.58     20047258    6.983   1728.825398
342.   Malawi       2022   66295.34     20568728    6.934   1820.452233
343.   Mozambique   2004   63947.57     19840313   14.073   678.3340608
344.   Mozambique   2005   65443.92     20304569   13.753   724.7475274
345.   Mozambique   2006   66999.13     20787087   13.399   800.3553392
346.   Mozambique   2007   68612.45     21287635    12.93   862.2761662
347.   Mozambique   2008   70325.22     21819036   12.536   915.0430299
348.   Mozambique   2009   72171.13     22391745   12.149   949.5807153
349.   Mozambique   2010   74129.13     22999235   11.902   997.0121092
350.   Mozambique   2011   76223.52     23649039   11.473   1058.669323
351.   Mozambique   2012   78443.63     24337846   10.995   1069.297846
352.   Mozambique   2013   80744.17     25051611   10.492   1104.926037
353.   Mozambique   2014   83119.17     25788475   10.005   1154.624788
354.   Mozambique   2015   85565.83     26547572    9.506   1303.371539
355.   Mozambique   2016   88110.58     27337105    9.049   1361.212187
356.   Mozambique   2017   90783.47     28166393     8.69   1264.653062
357.   Mozambique   2018   93530.37     29018644    8.337   1307.330596
358.   Mozambique   2019   96320.73     29884380     8.04   1370.398679
359.   Mozambique   2020    99219.3     30783688    8.004   1393.756781
360.   Mozambique   2021   102197.8     31707800    8.793   1440.459681
361.   Mozambique   2022   105254.8     32656246    8.611   1565.549233
362.   Myanmar      2004     151708     47068772    9.736   1591.721509
363.   Myanmar      2005   152899.2     47438365    9.628   1849.300795
364.   Myanmar      2006   154016.9     47785135    9.562   2139.296058
365.   Myanmar      2007   155112.5     48125043    9.409   2442.585217
366.   Myanmar      2008     155969     48390794   12.149   2728.978762
367.   Myanmar      2009   156838.2     48660459    9.193   3017.760519
368.   Myanmar      2010   158011.1     49024382    9.214   3323.277314
369.   Myanmar      2011   159285.7     49419820    9.026   3552.469936
370.   Myanmar      2012   160631.7     49837446    9.023   3816.000063
371.   Myanmar      2013   162002.2     50262658    8.799   4112.077289
372.   Myanmar      2014   163352.6     50681634    8.804   4342.900576
373.   Myanmar      2015   164665.8     51089056    8.673   4424.491426
374.   Myanmar      2016   165976.5     51495696    8.753   4425.452133
375.   Myanmar      2017   167263.3     51894938    8.808   4671.022595
376.   Myanmar      2018   168479.4     52272246    8.622   5539.547526
377.   Myanmar      2019     169667     52640713    8.692   6055.416906
378.   Myanmar      2020   170878.2     53016522    8.819   5697.288038
379.   Myanmar      2021   172072.7     53387102    9.769    5138.88725
380.   Myanmar      2022   173264.2     53756788    8.749   5682.541332
381.   Namibia      2004    6259.55      1942082   13.199   6488.704493
382.   Namibia      2005   6338.516      1966582   12.954   6779.413455
383.   Namibia      2006    6420.08      1991888   12.537   7393.542256
384.   Namibia      2007   6506.176      2018600   12.046   7902.759054
385.   Namibia      2008   6597.893      2047056    11.61   8158.708564
386.   Namibia      2009   6695.676      2077394   11.249    8116.40286
387.   Namibia      2010   6800.156      2109810   11.062   8581.068468
388.   Namibia      2011   6917.848      2146325   10.861   9061.271374
389.   Namibia      2012   7073.618      2194654   10.372   9481.204915
390.   Namibia      2013   7260.088      2252508     9.91   9910.385739
391.   Namibia      2014   7452.282      2312138    9.427   10684.80813
392.   Namibia      2015   7652.637      2374300    9.077   10885.84745
393.   Namibia      2016   7861.859      2439213    8.686   10708.80017
394.   Namibia      2017   8079.871      2506853     8.47   10483.08463
395.   Namibia      2018   8307.013      2577326    8.403   10556.80625
396.   Namibia      2019   8542.842      2650494    8.248   10410.13053
397.   Namibia      2020   8795.108      2728762    8.475   10254.79841
398.   Namibia      2021   9058.714      2810548   10.709   10785.08343
399.   Namibia      2022   9313.707      2889662   11.457   11987.41303
400.   Nigeria      2004   454642.8    141057045   16.271   3208.967185
401.   Nigeria      2005     467407    145017254   15.883   3429.047603
402.   Nigeria      2006   480493.1    149077334   15.561   3649.262566
403.   Nigeria      2007   493997.7    153267261   15.322   3888.373847
404.   Nigeria      2008   507946.5    157595014   15.161   4117.857641
405.   Nigeria      2009   522303.7    162049464    14.82   4355.811522
406.   Nigeria      2010   537108.9    166642886   14.628    4632.79413
407.   Nigeria      2011   552375.8    171379598   14.336   4843.604555
408.   Nigeria      2012   567914.5    176200624   14.193   4907.697163
409.   Nigeria      2013   583542.8    181049443   14.004   5153.786041
410.   Nigeria      2014   599166.8    185896915   13.892   5442.992611
411.   Nigeria      2015     614557    190671878   13.803   5377.747836
412.   Nigeria      2016   629937.1    195443700   13.638   5202.731561
413.   Nigeria      2017   645443.1    200254578   13.435   5171.432645
414.   Nigeria      2018   660540.8    204938755   13.239   5251.028231
415.   Nigeria      2019   675195.9    209485641   12.989   5525.443784
416.   Nigeria      2020   689733.9    213996181   12.997   5499.384691
417.   Nigeria      2021   704344.6    218529286   13.083   5623.664078
418.   Nigeria      2022   719240.6    223150896   12.431   6069.187109
419.   Papua Ne..   2004   20283.59      6293166    7.559   2198.657287
420.   Papua Ne..   2005   20946.42      6498818    7.563   2335.159671
421.   Papua Ne..   2006   21621.34      6708217    7.532   2458.193188
422.   Papua Ne..   2007   22307.38      6921066    7.501   2638.394095
423.   Papua Ne..   2008   23006.54      7137988    7.287   2599.773043
424.   Papua Ne..   2009   23718.53      7358890    7.215   2709.831725
425.   Papua Ne..   2010   24441.73      7583269    7.134   2931.189016
426.   Papua Ne..   2011   25161.67      7806637    6.951   2938.249398
427.   Papua Ne..   2012   25870.46      8026545    6.891   3046.531526
428.   Papua Ne..   2013   26576.59      8245627    6.825   3131.420096
429.   Papua Ne..   2014   27280.92      8464153    6.734   3524.061402
430.   Papua Ne..   2015   27983.63      8682174    6.585   3695.552329
431.   Papua Ne..   2016   28683.03      8899169     6.57   3839.508496
432.   Papua Ne..   2017   29378.02      9114796    6.508   3950.683432
433.   Papua Ne..   2018   30069.15      9329227    6.532   3937.277349
434.   Papua Ne..   2019   30756.51      9542486    6.468   4089.255587
435.   Papua Ne..   2020   31424.19      9749640    6.402   3926.781321
436.   Papua Ne..   2021   32068.16      9949437    6.683   3992.941221
437.   Papua Ne..   2022   32690.81     10142619    6.459   4409.232631
438.   Rwanda       2004   28417.68      8816844   11.546   834.6454993
439.   Rwanda       2005   29183.77      9054528   10.546   917.0864666
440.   Rwanda       2006   29972.52      9299246    9.803   1005.444317
441.   Rwanda       2007   30780.51      9549933    9.186   1081.970925
442.   Rwanda       2008   31600.26      9804268     8.64   1193.468707
443.   Rwanda       2009   32425.91     10060432    8.284   1242.611736
444.   Rwanda       2010   33254.64     10317554    8.068   1315.221836
445.   Rwanda       2011   34080.34     10573733    7.814   1412.480043
446.   Rwanda       2012      34906     10829901    7.604   1453.125355
447.   Rwanda       2013   35755.09     11093340    7.285   1511.150562
448.   Rwanda       2014   36629.89     11364757    6.953   1677.023634
449.   Rwanda       2015   37517.11     11640022    6.724   1780.292392
450.   Rwanda       2016   38416.88     11919183     6.49   1864.556869
451.   Rwanda       2017   39328.62     12202060    6.367   1963.392541
452.   Rwanda       2018   40250.22     12487996    6.227   2118.064338
453.   Rwanda       2019   41178.82     12776103    6.137   2325.314909
454.   Rwanda       2020   42112.67     13065837    5.998   2270.903332
455.   Rwanda       2021   43045.51     13355260     6.28    2709.45783
456.   Rwanda       2022   43998.81     13651030    5.847    3065.14536
457.   South Af..   2004   157904.9     48991420   12.947   9800.745355
458.   South Af..   2005     159512     49490032   13.177   10541.35199
459.   South Af..   2006   161150.1     49998277   13.234   11365.31898
460.   South Af..   2007   162859.3     50528584   12.973   12174.90907
461.   South Af..   2008   164748.1     51114598   12.543   12661.19878
462.   South Af..   2009   166726.9     51728516   11.947   12395.05511
463.   South Af..   2010   168710.8     52344051   11.257   12773.73806
464.   South Af..   2011   170809.5     52995205   10.592   13281.50389
465.   South Af..   2012   173347.3     53782566    9.993    13142.5888
466.   South Af..   2013   176235.9     54678792     9.77   13569.25285
467.   South Af..   2014   179188.4     55594838    9.432   13570.14971
468.   South Af..   2015   182826.4     56723537    9.259    13600.6168
469.   South Af..   2016     184554     57259551    8.924   13719.94319
470.   South Af..   2017   185764.6     57635162    8.802   13979.52387
471.   South Af..   2018   188916.3     58613001    8.765   13643.77414
472.   South Af..   2019   192058.5     59587885    8.614   13706.71382
473.   South Af..   2020   195199.4     60562381    9.425   13062.67277
474.   South Af..   2021   198229.8     61502603   11.432   14172.54425
475.   South Af..   2022   201052.7     62378410   12.032   15330.76927
476.   Tanzania     2004   122831.8     38109668   11.275   1472.456055
477.   Tanzania     2005   126288.3     39182076   10.879   1587.606689
478.   Tanzania     2006     129875     40294902   10.522   1695.308716
479.   Tanzania     2007   133551.3     41435512   10.154   1807.742554
480.   Tanzania     2008   137210.3     42570728    9.825   1895.012207
481.   Tanzania     2009   140637.3     43633982     9.41   1957.633789
482.   Tanzania     2010   144261.7     44758488     8.88   2053.252441
483.   Tanzania     2011   148357.8     46029367    8.435   2193.044434
484.   Tanzania     2012   152692.6     47374260    7.992     2065.5271
485.   Tanzania     2013   157365.7     48824128    7.615   2157.239014
486.   Tanzania     2014   162288.6     50351512    7.252   2200.939453
487.   Tanzania     2015   167669.4     52020962    6.959   2294.264404
488.   Tanzania     2016   173480.9     53824013     6.65   2408.841309
489.   Tanzania     2017   179375.5     55652890    6.402   2445.506348
490.   Tanzania     2018   185126.4     57437145    6.177    2697.20166
491.   Tanzania     2019   190727.4     59174891    6.012   2947.126953
492.   Tanzania     2020   196522.2     60972798    6.179   3251.588379
493.   Tanzania     2021   202509.5     62830412    6.222   3451.448242
494.   Tanzania     2022   208573.5     64711821    5.959   3751.166748
495.   Uganda       2004   87522.07     27154515   12.571   1405.679323
496.   Uganda       2005   90128.84     27963289   11.637   1497.403799
497.   Uganda       2006   92806.33     28794003    10.77   1660.923712
498.   Uganda       2007   95576.36     29653430   10.157   1795.966102
499.   Uganda       2008   98432.43     30539553    9.687   1932.587585
500.   Uganda       2009   101370.6     31451151    9.229   2017.086353
501.   Uganda       2010   104399.2     32390802    8.832   2094.733277
502.   Uganda       2011   107505.5     33354544    8.319   2271.727824
503.   Uganda       2012   110673.4     34337438    7.843   2035.749515
504.   Uganda       2013   113895.3     35337042     7.35   2048.561428
505.   Uganda       2014   117306.9     36395539     6.99   2137.474175
506.   Uganda       2015   120968.1     37531446    6.648   2193.125573
507.   Uganda       2016   125054.1     38799152    6.368   2168.139316
508.   Uganda       2017   129496.3     40177388    6.169   2160.662674
509.   Uganda       2018   133971.4     41565831    5.939   2314.866388
510.   Uganda       2019   138592.7     42999637    5.823   2444.248287
511.   Uganda       2020   143290.4     44457152    5.852   2535.215707
512.   Uganda       2021   147976.1     45910930    5.877   2688.266835
513.   Uganda       2022   152494.3     47312720    5.579    2920.66477
514.   Ukraine      2004   154643.4     47979507       16   6737.595703
515.   Ukraine      2005   153373.6     47585556     16.6   7214.943359
516.   Ukraine      2006   152384.6     47278694     16.2   8054.832031
517.   Ukraine      2007   151688.7     47062798     16.4   9006.356445
518.   Ukraine      2008   150903.5     46819175     16.3   9436.793945
519.   Ukraine      2009   150273.5     46623731     15.3   8093.634277
520.   Ukraine      2010     149733     46456003     15.2   8561.155273
521.   Ukraine      2011   149255.4     46307853     14.5   9246.769531
522.   Ukraine      2012   148940.2     46210056     14.5   9681.522461
523.   Ukraine      2013   148671.8     46126770     14.6   11056.54199
524.   Ukraine      2014   148174.2     45972380     14.7   10664.62402
525.   Ukraine      2015   147569.9     45784896     14.9   10064.84277
526.   Ukraine      2016   147028.2     45616832     14.7    11011.9707
527.   Ukraine      2017   146445.5     45436042     14.5   11686.85742
528.   Ukraine      2018   145713.4     45208907     14.8   12708.79883
529.   Ukraine      2019     144903     44957458     14.7   14380.86133
530.   Ukraine      2020   144008.7     44680014     15.9    15716.8418
531.   Ukraine      2021   142779.5     44298640     18.5    18040.3457
532.   Ukraine      2022   132304.8     41048766     21.4   16080.22852
533.   Vietnam      2004   258941.6     80338972    5.835   3458.203099
534.   Vietnam      2005   261356.8     81088313    5.865   3797.483499
535.   Vietnam      2006   264836.4     82167897    5.927   4147.359784
536.   Vietnam      2007   269559.8     83633375    5.966   4519.785666
537.   Vietnam      2008   274531.2     85175788    6.066   4820.216435
538.   Vietnam      2009   278670.4     86460018    6.117   5059.391788
539.   Vietnam      2010   281877.8     87455152    6.193   5391.940968
540.   Vietnam      2011   285143.4     88468314    6.195   5793.929889
541.   Vietnam      2012     288502     89510356    6.275   6391.723998
542.   Vietnam      2013   291927.3     90573104    6.327   6781.267213
543.   Vietnam      2014   295493.6     91679578    6.373   7332.395857
544.   Vietnam      2015   299179.8     92823254    6.444   7724.161017
545.   Vietnam      2016     302973     94000117    6.495   8453.065219
546.   Vietnam      2017   306766.2     95176977    6.577   9281.283364
547.   Vietnam      2018   310183.8     96237319    6.663   10149.49816
548.   Vietnam      2019   313202.1     97173776    6.706   11190.15319
549.   Vietnam      2020   316120.3     98079191    6.169    11781.1483
550.   Vietnam      2021     318879     98935098    7.317   12230.25962
551.   Vietnam      2022     321282     99680655    6.831   14051.24877
552.   Zambia       2004   36544.29     11338198    13.45   2095.691684
553.   Zambia       2005   37771.08     11718819   12.659   2242.268492
554.   Zambia       2006   39094.92     12129553   11.933   2409.374214
555.   Zambia       2007   40498.69     12565086   11.351    2588.26804
556.   Zambia       2008    41969.2     13021324    10.67   2743.479765
557.   Zambia       2009   43481.05     13490390    9.961   2909.626528
558.   Zambia       2010   45012.69     13965594    9.219   3136.633418
559.   Zambia       2011   46534.65     14437796    8.763   3267.408278
560.   Zambia       2012   48068.31     14913629    8.267   3361.721331
561.   Zambia       2013   49632.71     15398997    7.788   3514.718488
562.   Zambia       2014   51232.39     15895315    7.408   3475.759629
563.   Zambia       2015   52856.12     16399089    7.196   3368.460312
564.   Zambia       2016    54517.1     16914423    6.941   3342.331991
565.   Zambia       2017   56215.34     17441320    6.809   3419.717835
566.   Zambia       2018   57930.84     17973569    6.748   3468.844121
567.   Zambia       2019    59672.2     18513839    6.571    3385.78657
568.   Zambia       2020   61430.58     19059395    6.602   3179.265411
569.   Zambia       2021   63184.64     19603607    6.973   3526.507418
570.   Zambia       2022    64955.2     20152938    6.716   3864.894367
571.   Zimbabwe     2004   39856.68     12365896   16.989   1710.908686
572.   Zimbabwe     2005   40235.51     12483432    16.82   1655.085401
573.   Zimbabwe     2006   40728.69     12636445   16.436   1632.944064
574.   Zimbabwe     2007   41268.93     12804058    16.27   1600.327234
575.   Zimbabwe     2008    41768.8     12959150   15.604   1332.275083
576.   Zimbabwe     2009    42360.7     13142790   14.804   1486.281846
577.   Zimbabwe     2010   43049.66     13356548   13.281    1804.29539
578.   Zimbabwe     2011   43819.59     13595424   11.857   2080.611511
579.   Zimbabwe     2012   44536.61     13817887   10.691   2366.176012
580.   Zimbabwe     2013   45168.09     14013808    9.787   2558.994062
581.   Zimbabwe     2014   45791.92     14207358    9.123   2618.240859
582.   Zimbabwe     2015   46409.64     14399013    8.774     2692.2907
583.   Zimbabwe     2016    47058.4     14600294    8.441   2825.357165
584.   Zimbabwe     2017    47742.3     14812482    8.266   2435.794358
585.   Zimbabwe     2018   48457.74     15034452    7.972   2611.326409
586.   Zimbabwe     2019   49221.34     15271368    8.043   2711.529334
587.   Zimbabwe     2020   50044.91     15526888    8.132   2784.872395
588.   Zimbabwe     2021   50916.19     15797210    9.057   3276.267753
589.   Zimbabwe     2022   51792.38     16069056    8.943   3660.835501
```

**S11. Country Year Results through 2022 – Excluding India**

```
       country      year   lives saved population    acm   gdp_pc_cu~t
  1.   Angola       2004   60852.73    18600423   15.151   4224.139738
  2.   Angola       2005   63112.54    19291161   14.497   4836.226882
  3.   Angola       2006   65481.55    20015279   13.943   5364.965087
  4.   Angola       2007   67978.68    20778561   13.133    6057.77173
  5.   Angola       2008   70596.25    21578655   12.478   6616.768779
  6.   Angola       2009   73331.68    22414773   11.788   6471.347922
  7.   Angola       2010   76210.84    23294825   11.177   6587.408285
  8.   Angola       2011   79232.23    24218352    10.63     6700.1141
  9.   Angola       2012    82369.8    25177394   10.031   7351.760767
 10.   Angola       2013   85602.87    26165620    9.607   7566.423051
 11.   Angola       2014   88858.58    27160769    9.181   7999.826851
 12.   Angola       2015   92120.43    28157798    8.826   7127.339573
 13.   Angola       2016   95474.69    29183070    8.555   6850.384625
 14.   Angola       2017   98915.63    30234839    8.242   6998.796187
 15.   Angola       2018   102391.1    31297155     7.99   7353.349988
 16.   Angola       2019   105919.4    32375632    7.811   7533.511854
 17.   Angola       2020     109438    33451132    7.821   6455.119982
 18.   Angola       2021   112975.5    34532429    8.009   7414.278958
 19.   Angola       2022   116582.8    35635029    7.863   7928.641498
 20.   Botswana     2004   5912.219     1807146   13.073   10299.53078
 21.   Botswana     2005   6019.301     1839877   12.568   10908.62423
 22.   Botswana     2006   6132.625     1874516   11.883    11958.7509
 23.   Botswana     2007   6252.643     1911201   10.935   12741.39038
 24.   Botswana     2008   6379.479     1949970   10.253   13139.72502
 25.   Botswana     2009   6512.642     1990673    9.719   11119.91449
 26.   Botswana     2010   6651.478     2033110     8.93   12141.48683
 27.   Botswana     2011   6783.731     2073535    8.738    12976.5292
 28.   Botswana     2012   6898.502     2108616    8.671   12091.68763
 29.   Botswana     2013   7003.408     2140682    8.157   12880.30526
 30.   Botswana     2014   7106.011     2172044    7.927   14417.01321
 31.   Botswana     2015    7208.18     2203273    7.435   13518.48486
 32.   Botswana     2016   7311.244     2234776    6.783   15416.58452
 33.   Botswana     2017    7415.84     2266747    6.308   14817.57128
 34.   Botswana     2018   7521.819     2299141    6.922   14805.29163
 35.   Botswana     2019   7629.591     2332083    6.974    14889.8073
 36.   Botswana     2020   7740.207     2365894     6.95   14208.27016
 37.   Botswana     2021   7856.501     2401441    9.461   16663.86854
 38.   Botswana     2022   7982.297     2439892    6.897   18516.19134
 39.   Burundi      2004   23917.38     7310656   13.369   566.1589076
 40.   Burundi      2005   24822.44     7587299   12.841   567.7661336
 41.   Burundi      2006   25732.75     7865547   12.366   595.2640856
 42.   Burundi      2007   26702.21     8161874   11.871   609.6934915
 43.   Burundi      2008   27826.86     8505637   11.316   625.4000522
 44.   Burundi      2009   29272.92     8947646    10.92   620.9027826
 45.   Burundi      2010   30675.77     9376444   10.538   630.4483917
 46.   Burundi      2011   31793.12     9717978   10.094   646.1031275
 47.   Burundi      2012   32948.15    10071028    9.625   656.8448261
 48.   Burundi      2013   34153.11    10439340    9.178   706.9897821
 49.   Burundi      2014   35332.34    10799786    8.798   745.4026654
 50.   Burundi      2015   36143.02    11047580    8.553   821.2462547
 51.   Burundi      2016   36770.74    11239451     8.18   795.7859958
 52.   Burundi      2017   37645.27    11506762    7.772   785.2333947
 53.   Burundi      2018    38799.1    11859446    7.648   777.4469722
 54.   Burundi      2019   40094.29    12255336    7.352   804.1653441
 55.   Burundi      2020   41277.62    12617036    7.562    812.055173
 56.   Burundi      2021   42417.59    12965482    7.474   864.2807327
 57.   Burundi      2022   43581.01    13321098    7.311    917.506035
 58.   Cambodia     2004   43331.17    13244731    7.273   1505.462904
 59.   Cambodia     2005    43967.4    13439202    7.042   1727.836497
 60.   Cambodia     2006   44621.14    13639028    6.809   1939.122101
 61.   Cambodia     2007   45284.43    13841770     6.63    2157.12361
 62.   Cambodia     2008   45977.05    14053480    6.418   2307.270331
 63.   Cambodia     2009    46707.7    14276810    6.127   2288.740553
 64.   Cambodia     2010   47440.26    14500726    6.121   2419.182169
 65.   Cambodia     2011   48166.08    14722584    5.947   2605.489544
 66.   Cambodia     2012   48894.02    14945086    5.863   2855.491165
 67.   Cambodia     2013   49630.52    15170208    5.817   3025.421459
 68.   Cambodia     2014   50371.74    15396772    5.756    3158.53082
 69.   Cambodia     2015   51112.68    15623250     5.81   3354.610833
 70.   Cambodia     2016   51863.68    15852803    5.779   3631.128525
 71.   Cambodia     2017   52585.29    16073372    5.775   3873.636032
 72.   Cambodia     2018   53243.37    16274522    5.874   4228.760962
 73.   Cambodia     2019   53919.87    16481304    5.947   4620.963392
 74.   Cambodia     2020   54718.69    16725474    6.228    4504.24716
 75.   Cambodia     2021   55532.76    16974305    6.838    4668.23778
 76.   Cambodia     2022   56276.78    17201724    6.785   5202.677725
 77.   Cameroon     2004    54342.5    16610487   12.565   2502.676474
 78.   Cameroon     2005   55860.87    17074594   12.408   2567.519485
 79.   Cameroon     2006   57417.53    17550407   12.104   2673.826075
 80.   Cameroon     2007   59051.64    18049896   11.886   2786.542161
 81.   Cameroon     2008   60766.47    18574056   11.583   2839.404759
 82.   Cameroon     2009   62532.86    19113974     11.3   2848.357705
 83.   Cameroon     2010   64345.61    19668066   10.992   2883.163197
 84.   Cameroon     2011   66191.56    20232304   10.641   2957.163456
 85.   Cameroon     2012      68080    20809529   10.235    3027.17972
 86.   Cameroon     2013   70019.54    21402376    9.833   3204.361753
 87.   Cameroon     2014   72170.66    22059893    9.548   3385.476353
 88.   Cameroon     2015   74472.28    22763414    9.179   3459.780244
 89.   Cameroon     2016   76732.12    23454161    8.886   3587.616119
 90.   Cameroon     2017    78938.6    24128601    8.594    3726.56739
 91.   Cameroon     2018   81156.02    24806383    8.379   3967.521705
 92.   Cameroon     2019   83445.18    25506095     8.16   4195.670175
 93.   Cameroon     2020   85749.88    26210558    8.419   4319.218871
 94.   Cameroon     2021      88057    26915758    8.582    4741.76177
 95.   Cameroon     2022   90402.77    27632771    8.225   5122.644393
 96.   Cote d'I..   2004   64106.57    19595000   13.465   2952.392093
 97.   Cote d'I..   2005   65655.52    20068458   13.117   3006.344803
 98.   Cote d'I..   2006   67220.47    20546802   12.753    3116.67755
 99.   Cote d'I..   2007   68803.11    21030558   12.364   3167.190469
100.   Cote d'I..   2008   70386.16    21514439   11.952   3311.302978
101.   Cote d'I..   2009   71967.98    21997940   11.529   3379.445487
102.   Cote d'I..   2010   73571.46    22488065   11.187   3578.218921
103.   Cote d'I..   2011   75187.41    22982000   10.927   3384.930979
104.   Cote d'I..   2012   76774.38    23467078   10.517   3508.457834
105.   Cote d'I..   2013   78320.84    23939775   10.144   3855.865463
106.   Cote d'I..   2014   80243.29    24527395    9.847   4345.616675
107.   Cote d'I..   2015   82595.38    25246342    9.543   4711.492844
108.   Cote d'I..   2016   85026.37    25989405    9.374   4862.918019
109.   Cote d'I..   2017   87460.31    26733372     9.17   5046.047689
110.   Cote d'I..   2018   89851.19    27464173    8.961   5327.824985
111.   Cote d'I..   2019   92235.63    28193009    8.728   5947.924089
112.   Cote d'I..   2020   94599.15    28915449    8.817   5978.822741
113.   Cote d'I..   2021   96968.71    29639736    9.001    6520.73116
114.   Cote d'I..   2022   99439.62    30395002    8.843   7234.226693
115.   Democrat..   2004   186472.6    56997741   13.465   474.0152567
116.   Democrat..   2005   192289.4    58775724   13.136   502.9551638
117.   Democrat..   2006   198309.7    60615908   12.777   528.9236978
118.   Democrat..   2007   204400.8    62477752   12.529   558.9917424
119.   Democrat..   2008   210659.1    64390664   12.275   586.1752581
120.   Democrat..   2009   217272.2    66412044    11.98   587.5585393
121.   Democrat..   2010   224309.3    68563038   11.653   616.6206195
122.   Democrat..   2011     231789    70849311   11.301   650.4366032
123.   Democrat..   2012   239658.2    73254618   11.166   652.2973377
124.   Democrat..   2013   247950.9    75789395   10.857    735.030028
125.   Democrat..   2014   256502.3    78403242   10.551   829.6507769
126.   Democrat..   2015   265114.1    81035531   10.396   885.1244428
127.   Democrat..   2016     274670    83956415   10.077   942.3002777
128.   Democrat..   2017   284900.1    87083398    9.865   1036.588358
129.   Democrat..   2018   294597.9    90047644    9.573   1105.628847
130.   Democrat..   2019   304084.8    92947442     9.38   1206.798113
131.   Democrat..   2020   314038.8    95989998    9.537   1174.385868
132.   Democrat..   2021   324373.5    99148932      9.7   1358.204717
133.   Democrat..   2022   334999.7   102396968    9.352   1533.743878
134.   Dominica..   2004   29585.29     9043127    6.089   7452.720836
135.   Dominica..   2005   29983.25     9164768    5.933   8299.451614
136.   Dominica..   2006   30373.88     9284168    5.873   9220.173495
137.   Dominica..   2007   30760.05     9402206    5.859   10044.55488
138.   Dominica..   2008   31155.07     9522948    5.831   10432.70056
139.   Dominica..   2009   31564.38     9648061    5.831   10458.95548
140.   Dominica..   2010   31982.14     9775755    5.821   11319.09197
141.   Dominica..   2011   32400.85     9903737     5.84   11760.64189
142.   Dominica..   2012   32816.81    10030882    5.802   11846.43857
143.   Dominica..   2013   33229.59    10157051    5.803   12484.10743
144.   Dominica..   2014   33638.74    10282115    5.827   13541.93457
145.   Dominica..   2015   34043.49    10405832    5.883   14886.77901
146.   Dominica..   2016   34441.84    10527592    5.964   16351.83448
147.   Dominica..   2017   34833.29    10647244    6.032   17075.24595
148.   Dominica..   2018   35220.27    10765531     6.06   18141.33826
149.   Dominica..   2019   35600.93    10881882    6.006   19743.40045
150.   Dominica..   2020   35986.26    10999664     6.46   19085.35362
151.   Dominica..   2021   36372.99    11117873    6.678   21923.30194
152.   Dominica..   2022   36735.96    11228821    6.068   24363.89337
153.   Eswatini     2004    3510.92     1073158   18.372   5494.454546
154.   Eswatini     2005   3530.105     1079022   18.845   5972.358545
155.   Eswatini     2006   3547.817     1084436   18.174   6490.031863
156.   Eswatini     2007   3568.326     1090705   17.861   6921.234365
157.   Eswatini     2008   3592.494     1098092   17.525   7074.295716
158.   Eswatini     2009   3615.594     1105153   17.283   7196.207957
159.   Eswatini     2010    3636.85     1111650   15.911   7525.387057
160.   Eswatini     2011   3657.078     1117833   14.926   7814.518442
161.   Eswatini     2012   3676.805     1123863   13.946   8076.337181
162.   Eswatini     2013    3696.85     1129990   13.019   8567.039897
163.   Eswatini     2014   3717.491     1136299   12.193   8715.031268
164.   Eswatini     2015   3738.799     1142812   11.202   8792.214728
165.   Eswatini     2016   3760.702     1149507    10.45   8750.877727
166.   Eswatini     2017   3789.125     1158195    9.936   8519.584386
167.   Eswatini     2018   3824.242     1168929    9.324   8640.083598
168.   Eswatini     2019    3860.05     1179874    8.923   9083.437861
169.   Eswatini     2020   3902.106     1192729    9.603    9077.23198
170.   Eswatini     2021   3947.463     1206593   11.356   10202.50961
171.   Eswatini     2022   3987.782     1218917   11.883   10887.00387
172.   Ethiopia     2004   248892.5    76077206   12.745   563.5755313
173.   Ethiopia     2005   256385.3    78367470   12.226   631.7459772
174.   Ethiopia     2006   264027.8    80703490   11.539   701.6694792
175.   Ethiopia     2007   271878.7    83103218   10.807   780.6604652
176.   Ethiopia     2008   279862.8    85543668   10.143    856.873946
177.   Ethiopia     2009   287923.8    88007636    9.485   912.1172452
178.   Ethiopia     2010   296203.8    90538514    9.001   1010.166552
179.   Ethiopia     2011   304747.4    93149970     8.52   1114.045972
180.   Ethiopia     2012   313413.1    95798767    8.119   1195.794561
181.   Ethiopia     2013   322050.1    98438751    7.734   1270.347236
182.   Ethiopia     2014   330807.6   101115610    7.502   1505.005338
183.   Ethiopia     2015   339809.4   103867134    7.208   1654.847633
184.   Ethiopia     2016   349136.8   106718162     6.93   1882.687154
185.   Ethiopia     2017   358782.4   109666481    6.767     2031.9573
186.   Ethiopia     2018   368589.6   112664152    6.569   2124.254622
187.   Ethiopia     2019   378643.9   115737383    6.439   2274.214139
188.   Ethiopia     2020   389048.4   118917671    6.616   2442.252484
189.   Ethiopia     2021   399585.9   122138588    6.776   2628.343464
190.   Ethiopia     2022   410204.5   125384287    6.497   2888.736492
191.   Ghana        2004   71543.84    21868298    9.476   2724.591856
192.   Ghana        2005   73445.09    22449438    9.247   2897.701465
193.   Ghana        2006   75379.45    23040702     9.14   3095.449715
194.   Ghana        2007   77347.56    23642279    9.026   3232.192189
195.   Ghana        2008   79335.59    24249946    8.905   3504.585391
196.   Ghana        2009   81340.15    24862664    8.706   3604.467427
197.   Ghana        2010   83343.43    25474995    8.626   3840.435212
198.   Ghana        2011   85372.12    26095088    8.417   4362.592882
199.   Ghana        2012   87463.24    26734268    8.238   4910.784396
200.   Ghana        2013   89596.07    27386193    8.092   5188.160056
201.   Ghana        2014   91740.26    28041592    7.817   5486.848997
202.   Ghana        2015   93881.42    28696068    7.773   5159.926452
203.   Ghana        2016   96042.88    29356742     7.46   4977.581864
204.   Ghana        2017   98174.67    30008354     7.39   5134.361772
205.   Ghana        2018   100233.3    30637585    7.373   5494.700504
206.   Ghana        2019   102266.1    31258945    7.143   5952.403657
207.   Ghana        2020   104323.5    31887809    7.448    5992.31759
208.   Ghana        2021   106387.4    32518665    7.627     6544.2382
209.   Ghana        2022     108450    33149152    7.585   7132.783653
210.   Guyana       2004   2487.786      760424    7.402   6690.320613
211.   Guyana       2005   2485.447      759709    7.414   6771.842021
212.   Guyana       2006   2481.057      758367    7.297    7351.74251
213.   Guyana       2007   2475.018      756521     7.16   8113.674855
214.   Guyana       2008    2467.26      754150    7.288   8441.615559
215.   Guyana       2009   2457.799      751258    7.321   8834.309495
216.   Guyana       2010   2446.918      747932    7.399   9353.126083
217.   Guyana       2011   2434.806      744230    7.455   10092.08249
218.   Guyana       2012   2433.943      743966    7.513   10659.99316
219.   Guyana       2013   2445.243      747420    7.538   11238.86276
220.   Guyana       2014   2457.331      751115    7.459   11191.23459
221.   Guyana       2015   2470.143      755031    7.431   11465.13618
222.   Guyana       2016   2483.412      759087    7.475   11584.05925
223.   Guyana       2017   2497.038      763252    7.472    12327.0682
224.   Guyana       2018    2569.87      785514    7.499   12538.21374
225.   Guyana       2019   2613.183      798753    7.444   13388.70608
226.   Guyana       2020   2608.109      797202    8.026   17036.28992
227.   Guyana       2021   2632.204      804567    9.987    23176.1332
228.   Guyana       2022    2645.81      808726    9.762   40337.59901
229.   Haiti        2004   29318.21     8961489   10.184   2308.923633
230.   Haiti        2005   29810.29     9111900    9.239   2413.960842
231.   Haiti        2006   30315.38     9266288    9.084   2490.223854
232.   Haiti        2007   30820.97     9420826    8.937   2634.138229
233.   Haiti        2008   31326.17     9575247    8.853    2711.80526
234.   Haiti        2009   31834.54     9730638    8.695   2843.112786
235.   Haiti        2010   32201.75     9842880   17.738   2684.072674
236.   Haiti        2011   32566.31     9954312    8.762   2846.881182
237.   Haiti        2012   33070.88    10108539    8.486   2819.336533
238.   Haiti        2013   33570.34    10261206    8.376   3030.979635
239.   Haiti        2014   34066.09    10412740    8.243   3064.069363
240.   Haiti        2015   34560.16    10563757    8.181   3014.229685
241.   Haiti        2016   35051.19    10713849    8.161   3240.598619
242.   Haiti        2017   35540.93    10863543    8.014   3325.313579
243.   Haiti        2018      36028    11012421    7.998   3182.441427
244.   Haiti        2019   36512.24    11160438    7.953   3220.942957
245.   Haiti        2020   36991.08    11306801    8.176    3085.90305
246.   Haiti        2021   37451.61    11447569    8.676   3114.963542
247.   Haiti        2022   37901.22    11584996     8.48   3239.319005
248.   Indonesia    2004   745679.8   227926649    7.995   5750.204743
249.   Indonesia    2005   755314.6   230871650    7.336    6189.56886
250.   Indonesia    2006   765391.1   233951652    7.323    6644.53394
251.   Indonesia    2007   775567.9   237062338    7.314   7162.984355
252.   Indonesia    2008   785695.3   240157903     7.41   7639.919098
253.   Indonesia    2009   795713.3   243220028    7.386   7941.243696
254.   Indonesia    2010   805807.1   246305322    7.406   8431.821765
255.   Indonesia    2011   816160.7   249470032    7.456    9022.72138
256.   Indonesia    2012   826722.9   252698525    7.494   9624.588232
257.   Indonesia    2013   837041.3   255852467    7.457   9966.382935
258.   Indonesia    2014   846937.6   258877399     7.43   10168.67606
259.   Indonesia    2015   856496.7   261799249     7.46   10132.31608
260.   Indonesia    2016   865749.3   264627418    7.529   10371.44233
261.   Indonesia    2017   874645.4   267346658    7.579    10802.7126
262.   Indonesia    2018   883168.5   269951846    7.496   11494.94485
263.   Indonesia    2019   891470.3   272489381    7.539   12115.70207
264.   Indonesia    2020   899078.3   274814866    8.964   11856.94333
265.   Indonesia    2021   905435.6   276758053   10.065   12897.10314
266.   Indonesia    2022   912215.8   278830529     9.59   14445.64316
267.   Kenya        2004   113567.8    34713455     9.61   2061.660295
268.   Kenya        2005     117111    35796484    9.147   2185.854575
269.   Kenya        2006   120734.3    36904008    8.748   2328.789645
270.   Kenya        2007   124438.9    38036349    8.457     2481.0581
271.   Kenya        2008   128266.6    39206351    8.087   2460.342681
272.   Kenya        2009   132200.8    40408891    7.718   2482.775173
273.   Kenya        2010     136093    41598566     7.57   2640.012483
274.   Kenya        2011   139887.6    42758461    7.428    2758.24151
275.   Kenya        2012     143584    43888303    7.453   2857.851916
276.   Kenya        2013   147177.8    44986791    7.399   3119.877367
277.   Kenya        2014   150660.9    46051440    7.304   3375.584568
278.   Kenya        2015   154053.8    47088526    7.361   3696.936075
279.   Kenya        2016   157492.7    48139687     7.34   3936.689762
280.   Kenya        2017   160954.3    49197766    7.294   4198.553356
281.   Kenya        2018   164256.5    50207107    7.305   4433.975323
282.   Kenya        2019     167514    51202828     7.29    4710.50374
283.   Kenya        2020   170833.1    52217334    7.456    4813.92014
284.   Kenya        2021   174110.7    53219166    8.056   5360.361903
285.   Kenya        2022   177491.2    54252460     7.83   5902.091974
286.   Lesotho      2004   6448.702     1971129   20.162   1549.542644
287.   Lesotho      2005   6389.641     1953076   20.605   1660.177062
288.   Lesotho      2006   6363.422     1945062   20.884   1784.348091
289.   Lesotho      2007   6390.269     1953268   20.662   1903.006689
290.   Lesotho      2008   6433.692     1966541   20.417     2034.9595
291.   Lesotho      2009   6480.613     1980883   19.933   2007.595643
292.   Lesotho      2010   6530.891     1996251   18.644   2124.714206
293.   Lesotho      2011   6588.987     2014009   17.828   2252.036207
294.   Lesotho      2012   6655.548     2034354   17.019   2268.784986
295.   Lesotho      2013   6728.013     2056504   16.222   2516.924666
296.   Lesotho      2014   6805.173     2080089   15.519   2751.203047
297.   Lesotho      2015   6885.402     2104612   14.825   3014.721737
298.   Lesotho      2016   6969.842     2130422   14.126   2923.723188
299.   Lesotho      2017   7057.154     2157110   13.651   2602.522208
300.   Lesotho      2018   7143.828     2183603   13.259   2615.025922
301.   Lesotho      2019   7228.241     2209405   13.014   2549.535277
302.   Lesotho      2020   7314.355     2235727   12.858   2537.792082
303.   Lesotho      2021   7398.811     2261542   14.186   2516.977753
304.   Lesotho      2022   7479.187     2286110   14.069   2699.990941
305.   Malawi       2004   40897.14    12500737   15.541   1040.241089
306.   Malawi       2005   42007.16    12840031   14.633   1078.020519
307.   Malawi       2006   43180.58    13198701   13.951   1131.329257
308.   Malawi       2007   44416.07    13576343   13.496   1237.938778
309.   Malawi       2008   45724.94    13976418    12.96   1319.664038
310.   Malawi       2009   47096.94    14395788   12.183   1397.190323
311.   Malawi       2010   48505.93    14826463   11.412    1468.30528
312.   Malawi       2011   49940.71    15265022   10.608    1528.11656
313.   Malawi       2012   51395.11    15709577    9.783   1476.100989
314.   Malawi       2013   52874.64    16161816    9.081   1571.436944
315.   Malawi       2014   54378.66    16621538    8.376    1518.66973
316.   Malawi       2015   55896.83    17085587    8.005   1415.231394
317.   Malawi       2016   57441.51    17557739    7.541   1422.280238
318.   Malawi       2017   59018.34    18039715    7.149   1392.470808
319.   Malawi       2018   60616.06    18528081    6.955    1375.89008
320.   Malawi       2019   62244.23    19025752    6.611   1465.550469
321.   Malawi       2020   63906.64    19533888    6.706   1560.961347
322.   Malawi       2021   65586.17    20047258    6.983   1728.825398
323.   Malawi       2022    67292.2    20568728    6.934   1820.452233
324.   Mozambique   2004   64909.13    19840313   14.073   678.3340608
325.   Mozambique   2005   66427.98    20304569   13.753   724.7475274
326.   Mozambique   2006   68006.58    20787087   13.399   800.3553392
327.   Mozambique   2007   69644.16    21287635    12.93   862.2761662
328.   Mozambique   2008   71382.68    21819036   12.536   915.0430299
329.   Mozambique   2009   73256.34    22391745   12.149   949.5807153
330.   Mozambique   2010   75243.79    22999235   11.902   997.0121092
331.   Mozambique   2011   77369.67    23649039   11.473   1058.669323
332.   Mozambique   2012   79623.16    24337846   10.995   1069.297846
333.   Mozambique   2013    81958.3    25051611   10.492   1104.926037
334.   Mozambique   2014   84369.01    25788475   10.005   1154.624788
335.   Mozambique   2015   86852.45    26547572    9.506   1303.371539
336.   Mozambique   2016   89435.47    27337105    9.049   1361.212187
337.   Mozambique   2017   92148.55    28166393     8.69   1264.653062
338.   Mozambique   2018   94936.76    29018644    8.337   1307.330596
339.   Mozambique   2019   97769.08    29884380     8.04   1370.398679
340.   Mozambique   2020   100711.2    30783688    8.004   1393.756781
341.   Mozambique   2021   103734.5    31707800    8.793   1440.459681
342.   Mozambique   2022   106837.5    32656246    8.611   1565.549233
343.   Myanmar      2004   153989.2    47068772    9.736   1591.721509
344.   Myanmar      2005   155198.3    47438365    9.628   1849.300795
345.   Myanmar      2006   156332.8    47785135    9.562   2139.296058
346.   Myanmar      2007   157444.8    48125043    9.409   2442.585217
347.   Myanmar      2008   158314.3    48390794   12.149   2728.978762
348.   Myanmar      2009   159196.5    48660459    9.193   3017.760519
349.   Myanmar      2010   160387.1    49024382    9.214   3323.277314
350.   Myanmar      2011   161680.8    49419820    9.026   3552.469936
351.   Myanmar      2012   163047.1    49837446    9.023   3816.000063
352.   Myanmar      2013   164438.2    50262658    8.799   4112.077289
353.   Myanmar      2014   165808.9    50681634    8.804   4342.900576
354.   Myanmar      2015   167141.8    51089056    8.673   4424.491426
355.   Myanmar      2016   168472.2    51495696    8.753   4425.452133
356.   Myanmar      2017   169778.3    51894938    8.808   4671.022595
357.   Myanmar      2018   171012.7    52272246    8.622   5539.547526
358.   Myanmar      2019   172218.2    52640713    8.692   6055.416906
359.   Myanmar      2020   173447.7    53016522    8.819   5697.288038
360.   Myanmar      2021   174660.1    53387102    9.769    5138.88725
361.   Myanmar      2022   175869.5    53756788    8.749   5682.541332
362.   Namibia      2004   6353.673     1942082   13.199   6488.704493
363.   Namibia      2005   6433.826     1966582   12.954   6779.413455
364.   Namibia      2006   6516.617     1991888   12.537   7393.542256
365.   Namibia      2007   6604.007     2018600   12.046   7902.759054
366.   Namibia      2008   6697.104     2047056    11.61   8158.708564
367.   Namibia      2009   6796.356     2077394   11.249    8116.40286
368.   Namibia      2010   6902.408     2109810   11.062   8581.068468
369.   Namibia      2011    7021.87     2146325   10.861   9061.271374
370.   Namibia      2012   7179.982     2194654   10.372   9481.204915
371.   Namibia      2013   7369.256     2252508     9.91   9910.385739
372.   Namibia      2014    7564.34     2312138    9.427   10684.80813
373.   Namibia      2015   7767.708     2374300    9.077   10885.84745
374.   Namibia      2016   7980.076     2439213    8.686   10708.80017
375.   Namibia      2017   8201.365     2506853     8.47   10483.08463
376.   Namibia      2018   8431.923     2577326    8.403   10556.80625
377.   Namibia      2019   8671.298     2650494    8.248   10410.13053
378.   Namibia      2020   8927.357     2728762    8.475   10254.79841
379.   Namibia      2021   9194.927     2810548   10.709   10785.08343
380.   Namibia      2022   9453.755     2889662   11.457   11987.41303
381.   Nigeria      2004   461479.1   141057045   16.271   3208.967185
382.   Nigeria      2005   474435.3   145017254   15.883   3429.047603
383.   Nigeria      2006   487718.1   149077334   15.561   3649.262566
384.   Nigeria      2007   501425.8   153267261   15.322   3888.373847
385.   Nigeria      2008   515584.4   157595014   15.161   4117.857641
386.   Nigeria      2009   530157.4   162049464    14.82   4355.811522
387.   Nigeria      2010   545185.2   166642886   14.628    4632.79413
388.   Nigeria      2011   560681.8   171379598   14.336   4843.604555
389.   Nigeria      2012   576454.1   176200624   14.193   4907.697163
390.   Nigeria      2013   592317.4   181049443   14.004   5153.786041
391.   Nigeria      2014   608176.3   185896915   13.892   5442.992611
392.   Nigeria      2015   623797.9   190671878   13.803   5377.747836
393.   Nigeria      2016   639409.3   195443700   13.638   5202.731561
394.   Nigeria      2017   655148.5   200254578   13.435   5171.432645
395.   Nigeria      2018   670473.1   204938755   13.239   5251.028231
396.   Nigeria      2019   685348.6   209485641   12.989   5525.443784
397.   Nigeria      2020   700105.2   213996181   12.997   5499.384691
398.   Nigeria      2021   714935.6   218529286   13.083   5623.664078
399.   Nigeria      2022   730055.6   223150896   12.431   6069.187109
400.   Papua Ne..   2004   20588.58     6293166    7.559   2198.657287
401.   Papua Ne..   2005   21261.39     6498818    7.563   2335.159671
402.   Papua Ne..   2006   21946.46     6708217    7.532   2458.193188
403.   Papua Ne..   2007   22642.81     6921066    7.501   2638.394095
404.   Papua Ne..   2008   23352.48     7137988    7.287   2599.773043
405.   Papua Ne..   2009   24075.18     7358890    7.215   2709.831725
406.   Papua Ne..   2010   24809.26     7583269    7.134   2931.189016
407.   Papua Ne..   2011   25540.02     7806637    6.951   2938.249398
408.   Papua Ne..   2012   26259.47     8026545    6.891   3046.531526
409.   Papua Ne..   2013   26976.21     8245627    6.825   3131.420096
410.   Papua Ne..   2014   27691.14     8464153    6.734   3524.061402
411.   Papua Ne..   2015   28404.41     8682174    6.585   3695.552329
412.   Papua Ne..   2016   29114.33     8899169     6.57   3839.508496
413.   Papua Ne..   2017   29819.77     9114796    6.508   3950.683432
414.   Papua Ne..   2018   30521.29     9329227    6.532   3937.277349
415.   Papua Ne..   2019   31218.99     9542486    6.468   4089.255587
416.   Papua Ne..   2020   31896.71     9749640    6.402   3926.781321
417.   Papua Ne..   2021   32550.36     9949437    6.683   3992.941221
418.   Papua Ne..   2022   33182.37    10142619    6.459   4409.232631
419.   Rwanda       2004   28844.99     8816844   11.546   834.6454993
420.   Rwanda       2005   29622.59     9054528   10.546   917.0864666
421.   Rwanda       2006   30423.21     9299246    9.803   1005.444317
422.   Rwanda       2007   31243.35     9549933    9.186   1081.970925
423.   Rwanda       2008   32075.43     9804268     8.64   1193.468707
424.   Rwanda       2009   32913.49    10060432    8.284   1242.611736
425.   Rwanda       2010   33754.68    10317554    8.068   1315.221836
426.   Rwanda       2011   34592.79    10573733    7.814   1412.480043
427.   Rwanda       2012   35430.87    10829901    7.604   1453.125355
428.   Rwanda       2013   36292.73    11093340    7.285   1511.150562
429.   Rwanda       2014   37180.69    11364757    6.953   1677.023634
430.   Rwanda       2015   38081.24    11640022    6.724   1780.292392
431.   Rwanda       2016   38994.54    11919183     6.49   1864.556869
432.   Rwanda       2017   39919.99    12202060    6.367   1963.392541
433.   Rwanda       2018   40855.45    12487996    6.227   2118.064338
434.   Rwanda       2019   41798.02    12776103    6.137   2325.314909
435.   Rwanda       2020   42745.91    13065837    5.998   2270.903332
436.   Rwanda       2021   43692.77    13355260     6.28    2709.45783
437.   Rwanda       2022   44660.41    13651030    5.847    3065.14536
438.   South Af..   2004   160279.3    48991420   12.947   9800.745355
439.   South Af..   2005   161910.5    49490032   13.177   10541.35199
440.   South Af..   2006   163573.3    49998277   13.234   11365.31898
441.   South Af..   2007   165308.2    50528584   12.973   12174.90907
442.   South Af..   2008   167225.4    51114598   12.543   12661.19878
443.   South Af..   2009   169233.9    51728516   11.947   12395.05511
444.   South Af..   2010   171247.6    52344051   11.257   12773.73806
445.   South Af..   2011     173378    52995205   10.592   13281.50389
446.   South Af..   2012   175953.9    53782566    9.993    13142.5888
447.   South Af..   2013   178885.9    54678792     9.77   13569.25285
448.   South Af..   2014   181882.8    55594838    9.432   13570.14971
449.   South Af..   2015   185575.5    56723537    9.259    13600.6168
450.   South Af..   2016   187329.1    57259551    8.924   13719.94319
451.   South Af..   2017   188557.9    57635162    8.802   13979.52387
452.   South Af..   2018     191757    58613001    8.765   13643.77414
453.   South Af..   2019   194946.4    59587885    8.614   13706.71382
454.   South Af..   2020   198134.5    60562381    9.425   13062.67277
455.   South Af..   2021   201210.6    61502603   11.432   14172.54425
456.   South Af..   2022   204075.8    62378410   12.032   15330.76927
457.   Tanzania     2004   124678.8    38109668   11.275   1472.456055
458.   Tanzania     2005   128187.2    39182076   10.879   1587.606689
459.   Tanzania     2006   131827.9    40294902   10.522   1695.308716
460.   Tanzania     2007   135559.5    41435512   10.154   1807.742554
461.   Tanzania     2008   139273.5    42570728    9.825   1895.012207
462.   Tanzania     2009     142752    43633982     9.41   1957.633789
463.   Tanzania     2010   146430.9    44758488     8.88   2053.252441
464.   Tanzania     2011   150588.7    46029367    8.435   2193.044434
465.   Tanzania     2012   154988.6    47374260    7.992     2065.5271
466.   Tanzania     2013   159731.9    48824128    7.615   2157.239014
467.   Tanzania     2014   164728.9    50351512    7.252   2200.939453
468.   Tanzania     2015   170190.6    52020962    6.959   2294.264404
469.   Tanzania     2016   176089.5    53824013     6.65   2408.841309
470.   Tanzania     2017   182072.8    55652890    6.402   2445.506348
471.   Tanzania     2018   187910.1    57437145    6.177    2697.20166
472.   Tanzania     2019   193595.3    59174891    6.012   2947.126953
473.   Tanzania     2020   199477.3    60972798    6.179   3251.588379
474.   Tanzania     2021   205554.6    62830412    6.222   3451.448242
475.   Tanzania     2022   211709.8    64711821    5.959   3751.166748
476.   Uganda       2004   88838.12    27154515   12.571   1405.679323
477.   Uganda       2005   91484.08    27963289   11.637   1497.403799
478.   Uganda       2006   94201.83    28794003    10.77   1660.923712
479.   Uganda       2007   97013.51    29653430   10.157   1795.966102
480.   Uganda       2008   99912.53    30539553    9.687   1932.587585
481.   Uganda       2009   102894.9    31451151    9.229   2017.086353
482.   Uganda       2010     105969    32390802    8.832   2094.733277
483.   Uganda       2011     109122    33354544    8.319   2271.727824
484.   Uganda       2012   112337.6    34337438    7.843   2035.749515
485.   Uganda       2013   115607.9    35337042     7.35   2048.561428
486.   Uganda       2014   119070.8    36395539     6.99   2137.474175
487.   Uganda       2015   122787.1    37531446    6.648   2193.125573
488.   Uganda       2016   126934.5    38799152    6.368   2168.139316
489.   Uganda       2017   131443.5    40177388    6.169   2160.662674
490.   Uganda       2018   135985.9    41565831    5.939   2314.866388
491.   Uganda       2019   140676.7    42999637    5.823   2444.248287
492.   Uganda       2020     145445    44457152    5.852   2535.215707
493.   Uganda       2021   150201.2    45910930    5.877   2688.266835
494.   Uganda       2022   154787.3    47312720    5.579    2920.66477
495.   Ukraine      2004   156968.7    47979507       16   6737.595703
496.   Ukraine      2005   155679.9    47585556     16.6   7214.943359
497.   Ukraine      2006   154675.9    47278694     16.2   8054.832031
498.   Ukraine      2007   153969.6    47062798     16.4   9006.356445
499.   Ukraine      2008   153172.6    46819175     16.3   9436.793945
500.   Ukraine      2009   152533.2    46623731     15.3   8093.634277
501.   Ukraine      2010   151984.4    46456003     15.2   8561.155273
502.   Ukraine      2011   151499.8    46307853     14.5   9246.769531
503.   Ukraine      2012   151179.8    46210056     14.5   9681.522461
504.   Ukraine      2013   150907.3    46126770     14.6   11056.54199
505.   Ukraine      2014   150402.2    45972380     14.7   10664.62402
506.   Ukraine      2015   149788.9    45784896     14.9   10064.84277
507.   Ukraine      2016     149239    45616832     14.7    11011.9707
508.   Ukraine      2017   148647.6    45436042     14.5   11686.85742
509.   Ukraine      2018   147904.5    45208907     14.8   12708.79883
510.   Ukraine      2019   147081.8    44957458     14.7   14380.86133
511.   Ukraine      2020   146174.2    44680014     15.9    15716.8418
512.   Ukraine      2021   144926.5    44298640     18.5    18040.3457
513.   Ukraine      2022   134294.2    41048766     21.4   16080.22852
514.   Vietnam      2004   262835.2    80338972    5.835   3458.203099
515.   Vietnam      2005   265286.8    81088313    5.865   3797.483499
516.   Vietnam      2006   268818.7    82167897    5.927   4147.359784
517.   Vietnam      2007   273613.1    83633375    5.966   4519.785666
518.   Vietnam      2008   278659.2    85175788    6.066   4820.216435
519.   Vietnam      2009   282860.7    86460018    6.117   5059.391788
520.   Vietnam      2010   286116.3    87455152    6.193   5391.940968
521.   Vietnam      2011     289431    88468314    6.195   5793.929889
522.   Vietnam      2012   292840.1    89510356    6.275   6391.723998
523.   Vietnam      2013     296317    90573104    6.327   6781.267213
524.   Vietnam      2014   299936.9    91679578    6.373   7332.395857
525.   Vietnam      2015   303678.5    92823254    6.444   7724.161017
526.   Vietnam      2016   307528.7    94000117    6.495   8453.065219
527.   Vietnam      2017   311378.9    95176977    6.577   9281.283364
528.   Vietnam      2018   314847.9    96237319    6.663   10149.49816
529.   Vietnam      2019   317911.6    97173776    6.706   11190.15319
530.   Vietnam      2020   320873.7    98079191    6.169    11781.1483
531.   Vietnam      2021   323673.9    98935098    7.317   12230.25962
532.   Vietnam      2022     326113    99680655    6.831   14051.24877
533.   Zambia       2004    37093.8    11338198    13.45   2095.691684
534.   Zambia       2005   38339.03    11718819   12.659   2242.268492
535.   Zambia       2006   39682.78    12129553   11.933   2409.374214
536.   Zambia       2007   41107.66    12565086   11.351    2588.26804
537.   Zambia       2008   42600.28    13021324    10.67   2743.479765
538.   Zambia       2009   44134.86    13490390    9.961   2909.626528
539.   Zambia       2010   45689.53    13965594    9.219   3136.633418
540.   Zambia       2011   47234.38    14437796    8.763   3267.408278
541.   Zambia       2012    48791.1    14913629    8.267   3361.721331
542.   Zambia       2013   50379.02    15398997    7.788   3514.718488
543.   Zambia       2014   52002.76    15895315    7.408   3475.759629
544.   Zambia       2015    53650.9    16399089    7.196   3368.460312
545.   Zambia       2016   55336.86    16914423    6.941   3342.331991
546.   Zambia       2017   57060.64    17441320    6.809   3419.717835
547.   Zambia       2018   58801.93    17973569    6.748   3468.844121
548.   Zambia       2019   60569.47    18513839    6.571    3385.78657
549.   Zambia       2020    62354.3    19059395    6.602   3179.265411
550.   Zambia       2021   64134.73    19603607    6.973   3526.507418
551.   Zambia       2022   65931.91    20152938    6.716   3864.894367
552.   Zimbabwe     2004   40455.99    12365896   16.989   1710.908686
553.   Zimbabwe     2005   40840.52    12483432    16.82   1655.085401
554.   Zimbabwe     2006   41341.12    12636445   16.436   1632.944064
555.   Zimbabwe     2007   41889.47    12804058    16.27   1600.327234
556.   Zimbabwe     2008   42396.87    12959150   15.604   1332.275083
557.   Zimbabwe     2009   42997.66    13142790   14.804   1486.281846
558.   Zimbabwe     2010   43696.99    13356548   13.281    1804.29539
559.   Zimbabwe     2011   44478.49    13595424   11.857   2080.611511
560.   Zimbabwe     2012    45206.3    13817887   10.691   2366.176012
561.   Zimbabwe     2013   45847.27    14013808    9.787   2558.994062
562.   Zimbabwe     2014   46480.48    14207358    9.123   2618.240859
563.   Zimbabwe     2015    47107.5    14399013    8.774     2692.2907
564.   Zimbabwe     2016      47766    14600294    8.441   2825.357165
565.   Zimbabwe     2017   48460.19    14812482    8.266   2435.794358
566.   Zimbabwe     2018   49186.38    15034452    7.972   2611.326409
567.   Zimbabwe     2019   49961.47    15271368    8.043   2711.529334
568.   Zimbabwe     2020   50797.43    15526888    8.132   2784.872395
569.   Zimbabwe     2021    51681.8    15797210    9.057   3276.267753
570.   Zimbabwe     2022   52571.17    16069056    8.943   3660.835501
```
